# *k*-mer-based diversity scales with population size proxies more than nucleotide diversity in a meta-analysis of 98 plant species

**DOI:** 10.1093/evlett/qraf011

**Published:** 2025-06-12

**Authors:** Miles D Roberts, Emily B Josephs

**Affiliations:** Genetics and Genome Sciences Program, Michigan State University, East Lansing, MI, United States; Department of Plant Biology, Michigan State University, East Lansing, MI, United States; Ecology, Evolution, and Behavior Program, Michigan State University, East Lansing, MI, United States; Plant Resilience Institute, Michigan State University, East Lansing, MI, United States

**Keywords:** Lewontin’s paradox, k-mer, nucletide diversity, phylogenetic least squares, census size, neutral theory

## Abstract

A key prediction of neutral theory is that the level of genetic diversity in a population should scale with population size. However, as was noted by Richard Lewontin in 1974 and reaffirmed by later studies, the slope of the population size-diversity relationship in nature is much weaker than expected under neutral theory. We hypothesize that one contributor to this paradox is that current methods relying on single nucleotide polymorphisms (SNPs) called from aligning short reads to a reference genome underestimate levels of genetic diversity in many species. As a first step to testing this idea, we calculated nucleotide diversity (π) and k-mer-based metrics of genetic diversity across 112 plant species, amounting to over 205 terabases of DNA sequencing data from 27,488 individuals. After excluding 14 species with low coverage or no variant sites called, we compared how different diversity metrics correlated with proxies of population size that account for both range size and population density variation across species. We found that our population size proxies scaled anywhere from about 3 to over 20 times faster with k-mer diversity than nucleotide diversity after adjusting for evolutionary history, mating system, life cycle habit, cultivation status, and invasiveness. The relationship between k-mer diversity and population size proxies also remains significant after correcting for genome size, whereas the analogous relationship for nucleotide diversity does not. These results are consistent with the possibility that variation not captured by common SNP-based analyses explains part of Lewontin’s paradox in plants, but larger scale pangenomic studies are needed to definitively address this question.

Understanding the determinants of genetic diversity within populations is key to informing species conservation (Cole, 2003) and breeding efforts (Sanchez et al., 2023). However, most species have far less genetic diversity (commonly estimated as pairwise nucleotide diversity, π) than expected (Buffalo, 2021; Corbett-Detig et al., 2015; Frankham, 2012). If we assume that the vast majority of genetic variants are neutral, then the determinants of genetic diversity are encapsulated in neutral theory (Kimura, 1983): $E[\pi ]\approx 4N_{e}\mu$, where E[π] is the expected level of genetic diversity, $N_{e}$ is the effective size of a population, and μ is the mutation rate per base pair per generation. Mutations rates for single nucleotide polymorphisms (SNPs) and small indels vary relatively little across species (Cagan et al., 2022; Bergeron et al., 2023; Quiroz et al., 2023), while the total number of individuals in a species varies massively (Buffalo, 2021). Thus, under neutral theory, population size should be a strong determinant of genetic diversity and species with larger population sizes should be more diverse. However, even some of the most abundant species studied to date have low genetic diversity compared with neutral theory expectations. For example, *Drosophila simulans* has an estimated population size $>10^{14}$ and a diversity of π≈0.01, but an expected diversity of π>0.1 (Buffalo, 2021). This mismatch between expected and observed levels of neutral diversity across populations of varying size is known as Lewontin’s paradox, named after Richard Lewontin who first described the phenomenon (Lewontin, 1974).

The potential mechanisms underlying Lewontin’s paradox have been reviewed extensively (Leffler et al., 2012; Slotte, 2014; Ellegren and Galtier, 2016; Charlesworth and Jensen, 2022). Multiple selective and demographic processes likely contribute to Lewontin’s paradox; however, determining the relative importance of these processes remains a contentious area of research. The two most explored mechanisms are historic population size changes (i.e., demography, Charlesworth and Jensen (2022)) and linked selection—whereby fixation or purging of selected alleles causes the loss of linked neutral alleles (Kojima and Schaffer, 1964, 1967; Smith and Haigh, 1974; Charlesworth et al., 1993; Charlesworth, 1994). Linked selection is expected to reduce diversity more in regions of lower recombination and higher functional density (Slotte, 2014) and many studies have tested this hypothesis (Tenaillon et al., 2001; Hellmann et al., 2003; Nordborg et al., 2005; Roselius et al., 2005; Branca et al., 2011; Paape et al., 2012; Corbett-Detig et al., 2015; Silva-Junior and Grattapaglia, 2015; Wang et al., 2016; Phung et al., 2016; Mackintosh et al., 2019). These previous investigations often conclude that linked selection contributes to Lewontin’s paradox, but not all report significant results (e.g., Schmid et al. (2005); Roselius et al. (2005); Flowers et al. (2012); Wang et al. (2016)). It has been argued that studies focused on plant species especially tend to find weaker evidence for linked selection (Slotte, 2014). There is also both empirical and theoretical evidence that linked selection is unlikely to explain the entirety of Lewontin’s paradox, suggesting that demographic factors play an important role too (Coop, 2016; Buffalo, 2021; Charlesworth and Jensen, 2022).

There are three main types of demographic changes proposed to contribute to Lewontin’s paradox: contractions, expansions, and cyclical population size changes (Charlesworth and Jensen, 2022). Population contractions cause loss of diversity. Thus, if many species’ populations recently contracted (due to human activity, for example), then their contemporary diversity would be much lower than expected from their precontraction population sizes (Exposito-Alonso et al., 2022). Recent population expansions could cause a similar mismatch. Because it takes many generations for populations to accumulate diversity compared with the timescale of typical expansions, contemporary diversity levels for an expanded population would be much smaller than expected from a post-expansion population size (Peart et al., 2020; Charlesworth and Jensen, 2022). For a similar reason, species that have seasonal variation in their population sizes will also tend to have diversity levels closer to what one would expect based on their minimum size rather than their peak size (Wright, 1940). Studies investigating Lewontin’s paradox would ideally try to jointly infer these demographic histories alongside selective factors in natural populations. However, issues of model complexity and identifiability often prevent such joint estimation (Johri et al., 2020, 2022b,a), suggesting that further explorations of Lewontin’s paradox will require new approaches.

Two potential, but rarely explored, contributors to Lewontin’s paradox are that (1) current methods for estimating genetic diversity systematically underestimate the true levels of genetic diversity in most populations and (2) changes or corrections to the calculation of diversity could yield stronger correlations with population size. Lewontin’s original observations and earlier studies on the population size-diversity relationship were based on allozymes, which detect variants in protein sequences (Lewontin, 1974; Nei and Graur, 1984). More recent studies measure diversity using SNPs at more neutral fourfold degenerate sites (i.e., sites where mutations do not affect protein sequences) in DNA and generally observe greater within-species diversity and between-species divergence compared with allozymes (Li and Sadler, 1991; Makałowski and Boguski, 1998; Bazin et al., 2006; Piganeau and Eyre-Walker, 2009). However, current SNP-based methods are not perfect either and there is significant evidence that SNPs capture a biased and incomplete picture of genetic diversity. Calling SNPs typically requires aligning reads to a reference genome, meaning any SNPs in regions that are not present or highly diverged from the reference genome will be excluded from analysis and thus downwardly bias diversity estimates (Golicz et al., 2020; Buffalo, 2021). This downward bias is typically assumed to have little effect on the qualitative relationship between diversity and $N_{e}$ (Buffalo, 2021), but recent pangenomic studies have uncovered troves of nonreference variation across a variety of species (Ebler et al. (2022); Rice et al. (2023), reviewed in Bayer et al. (2020)). Sometimes, alignment errors in regions of nonreference structural variation will also create false positive SNP calls, which will also affect diversity estimates (e.g., Jaegle et al. (2023)). Finally, previous meta-analyses of population size and diversity data rely on scraping diversity estimates from previously published studies (Frankham (2012); Buffalo (2021), except see Corbett-Detig et al. (2015)). However, many studies report inaccurate SNP calls and deflated diversity estimates due to errors in the handling of missing genotype calls (Korunes and Samuk, 2021; Schmidt et al., 2021; Sopniewski and Catullo, 2024) and may filter genotype calls differently, making comparisons across species difficult. Overall, errors in diversity calculations and omission of diversity in genomic regions that are either difficult or impossible to align to could partially explain Lewontin’s paradox. Reanalyzing whole genome sequencing data with a common pipeline and applying correct calculations of nucleotide diversity would make diversity estimates across species more comparable and easier to interpret (Buffalo, 2021; Mirchandani et al., 2024).

One useful pangenomics tool for measuring nonreference variation that is readily applicable to common short-read datasets is the k-mer. k-mers are subsequences of length k derived from a larger sequence and they have a long history of use in computer science (Shannon, 1948), genome assembly (Turner et al., 2018), metagenomics (Benoit et al., 2016), and quantitative genetics (Rahman et al., 2018; Voichek and Weigel, 2020; Kim et al., 2020; Mehrab et al., 2021). Recent studies have also demonstrated the utility of k-mers for measuring heterozygosity and genetic differences between individuals (commonly referred to as “dissimilarity" measures, Ondov et al. (2016); Vurture et al. (2017); Ranallo-Benavidez et al. (2020); VanWallendael and Alvarez (2022); Roberts et al. (2024)). Typical analysis of k-mers involves only counting the presence/absence and/or frequencies of all k-mers in a set of reads, without aligning the reads to any reference, then deriving measures of genetic difference from such counts (Benoit et al., 2016). Avoiding alignment allows one to incorporate sequences that would otherwise be omitted for lack of alignment to a reference genome (Rahman et al., 2018; Voichek and Weigel, 2020; Wiersma et al., 2024).

We revisited Lewontin’s paradox in plants using k-mer-based measures of genetic difference and corrected π calculations, aiming to test whether the inclusion of nonreference variation or modifications to diversity calculations could increase the scaling between population size proxies and diversity. We compared how k-mer dissimilarity and typical SNP-based estimates of nucleotide diversity correlated with population size proxies across a large panel of plant species—all processed through the same bioinformatic pipeline. Our expectation was that if k-mers are better at capturing genomic variation than SNPs, k-mer dissimilarity would scale more rapidly with population size compared to nucleotide diversity.

## Methods

Our entire analysis is packaged as a snakemake workflow stored here: https://github.com/milesroberts-123/tajimasDacrossSpecies. This workflow includes the code to reproduce all of the steps individually explained below, along with instructions on how to run the code, and yaml files describing the exact configurations of software we used at each step. It also includes an example directed acyclic graph showing the order of steps a typical sample is processed through. The code detailing all initial, exploratory, and confirmatory data analyses as well as figure creation can be found as an R-markdown file in the github repository. We provide details on how we selected species with high quality, publicly available reference genomes, how we computed SNP calls, k-mer counts, and population size proxies in the supplementary material. The parameters for each software were kept constant across all datasets (except occasionally for the “–ploidy" parameter in GATK HaplotypeCaller) to ensure that variation in bioinformatic processing did not bias our results. All statistical analyses used R v4.2.2 (R Core Team, 2022) and all plots were made with ggplot2 (Wickham, 2016). Color palettes used in figure creation come from the scico R package (Pedersen and Crameri, 2023) to ensure color-blind accessibility. Table S1 contains citations for all studies that were used to label each species in our study with a genome size, mating system, ploidy level, cultivation status, and life-cycle habit.

### Statistical analysis

The ultimate goal of our statistical analyses was to estimate the effect of our population size proxies on measures of diversity, comparing the effects of using k-mer-based or nucleotide diversity. To do this, we used the caper R package (Orme et al., 2018) to implement a statistical approach similar to Whitney et al. (2010). We performed partial phylogenetic least squares regressions controlling for evolutionary history (using a phylogeny obtained from timetree.org, Kumar et al. (2017, 2022)), mating system (outcrossing vs. not outcrossing), cultivation status (wild vs. cultivated), and life cycle habit (annual vs. not annual). We did not include sequencing coverage as a covariate at this step because we would later exclude species with low coverage data from our analyses (see **Results**) and the relationship between coverage and diversity saturates at higher levels of coverage (Figure S3A, D, and G). We also did not include number of individuals as a covariate because this did not correlate with any of our diversity measures (Figure S3C, F, and I). Similar to Whitney et al. (2010), we also scaled the dependent variables to be unitless with a mean of zero and unit variance across species (using the scale() function in R) before performing regression to make slopes more comparable across models and account for the inherent differences in unit between nucleotide and k-mer diversity metrics. This approach can be summarized as follows:


scale(diversity)=β0+β1× log ⁡ 10(population size proxy)+β2×mating system+β3×cultivation status+β4×life cycle habit+ϵ,

where population size proxy refers to either Supplemental Equation 7 or its components (range size and plant height), diversity was estimated using either SNPs ($\log_{10}(\bar{\pi })$) or k-mers ($\bar{J_{D}}$ or $\bar{B_{D}}$), and the scale() function performs a z-transformation to make diversity unitless with a mean of zero and unit variance. We also constructed a separate set of models where we included genome size as a covariate;


scale(diversity)=β0+β1×log ⁡ 10(population size proxy)+β2×mating system+β3×cultivation status+β4×life cycle habit+β5×log ⁡ 10(genome size)+ϵ.

We controlled for genome size in a separate set of models because we had conflicting expectations on whether genome size would be a confounder or a mediator of the population size–diversity relationship. In other words, the effect of population size on diversity could act through genome size, since small populations may not experience strong enough selection to purge deleterious insertions (Lynch and Conery, 2003). Including genome size as a covariate in this case would artificially diminish the estimated effect of population size on diversity. Alternatively, genome size could fundamentally alter the mode of adaptation in plant species (Mei et al., 2018), making genome size a confounder of the population size–diversity relationship.

After constructing our models, we visualized the relationship between population size and diversity or genome size and diversity with partial regression plots, following methods from Riddell (1977) and Blomberg et al. (2012). Beginning with our initial phylogenetic least squares model


$$\begin{array}{ccc} y=X\beta +\epsilon , & & \end{array}$$ (1)

where y is a vector of diversity values, X is the design matrix, β is a vector of regression coefficients, and ϵ is a vector of residuals distributed normally about 0 with phylogenetic variance-covariance matrix Ω. We first performed Cholesky decomposition on Ω to get matrix C such that


$$\begin{array}{ccc} \Omega =CC^{T}. & & \end{array}$$ (2)

We then took the inverse matrix $C^{-1}$ and left-multiplied both sides of our regression equations to get


$$\begin{array}{ccc} C^{-1}y=C^{-1}X\beta +C^{-1}\epsilon , & & \end{array}$$ (3)

which we will rewrite as


$$\begin{array}{ccc} y^{*}=X^{*}\beta +\epsilon^{*}, & & \end{array}$$ (4)

where $y^{*}=C^{-1}y$, $X^{*}=C^{-1}X$, and $\epsilon^{*}=C^{-1}\epsilon$. In vector form, this equation is now


$$\begin{array}{ccc} y^{*}=\beta_{0}x_{0}^{*}+\beta_{1}x_{1}^{*}+\beta_{2}x_{2}^{*}+...+\beta_{n-1}x_{n-1}^{*}+\epsilon^{*}, & & \end{array}$$ (5)

where $\beta_{0}x_{0}^{*}$ is our intercept (Note that $x_{0}$ was initially a column of 1’s before being transformed by $C^{-1}$). After fitting this model to our data with the standard lm() function in R, we collected all terms besides the primary variable of interest, $x_{k}^{*}$ (which would be a population size proxy or genome size in our case) and subtracted them from both sides of the equation to get


$$\begin{array}{ccc} y^{*}-\underset{i\neq k}{\sum }\beta_{i}x_{i}^{*}=\beta_{k}x_{k}^{*}+\epsilon^{*}. & & \end{array}$$ (6)

We then plotted the values of $x_{k}^{*}$ against $y^{*}-\sum_{i\neq k}\beta_{i}x_{i}^{*}$, interpreting the slope ($\beta_{k}$) as the effect of the primary variable on the response, scaled for phylogenetic relationships and adjusted for the effects of confounding factors.

## Results

### Low diversity species explained by low mean coverage

**Figure 1:**
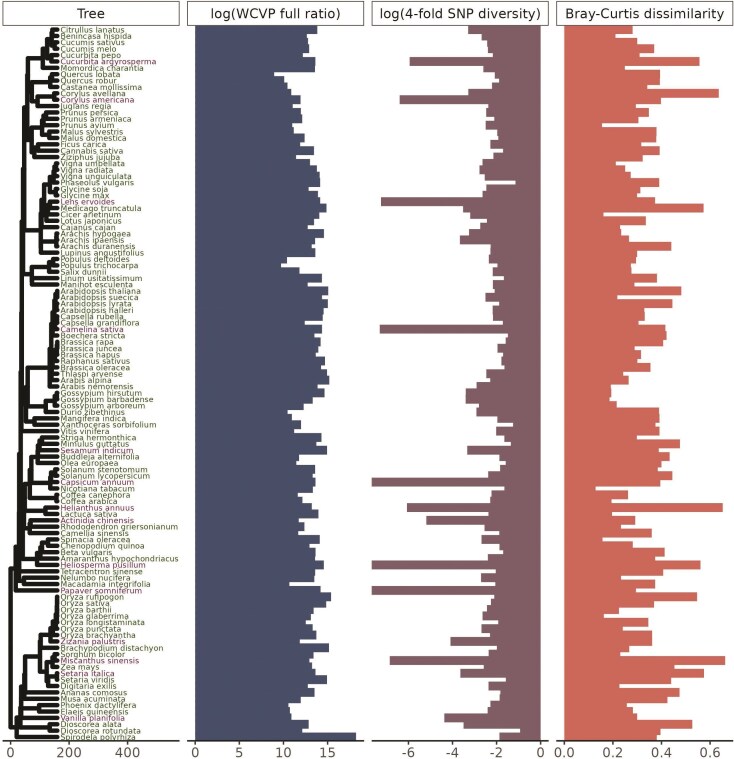
**Our study includes 112 plant species across a wide range of population sizes and diversity levels.** Species labeled in purple were considered outliers and omitted from downstream analyses (see Figure 2), but species labeled in green were retained. The phylogenetic tree is scaled in millions of years. The WCVP full ratio is a unitless population size proxy equal to the ratio of range area, estimated using WCVP range maps, to squared plant height and is log-transformed (base 10, see Supplemental Equation 7). Nucleotide diversity is genome-wide average diversity at fourfold degenerate sites, log-transformed (base 10, see Supplemental Equation 1). *Capsicum annuum*, *H. pusillum*, and *P. somniferum* had nucleotide diversity values of zero and so have bars at the plotting limit (log ⁡ (0)=−∞). Bray-Curtis dissimilarity is average pairwise Bray-Curtis dissimilarity across all pairs of individuals in a species’ sample (see Supplemental Equation 4).

There were 112 species in our initial dataset, each with estimates of population size proxies, nucleotide diversity, and k-mer diversity (Figure 1). Out of these 112 species, 102 were diploids, 9 were tetraploids, and 1 was hexaploid, with haploid genome sizes ranging from 105 Mb to 5.06 Gb (Table S1). These species were further broken down into 57 annual species vs. 55 not annual species (which were predominately perennial), 31 wild vs. 81 cultivated species, and 55 outcrossing vs. 57 not outcrossing species (which were predominantly selfing). Species classified as annual also tended to be classified as not outcrossing ($\chi^{2}=18.9,p=1.4\times 10^{-5}$, Figure S1C). However, cultivation status was independent of both life cycle habit ($\chi^{2}=4.07\times 10^{-31}$, *p* = 1, Figure S1A) and mating system ($\chi^{2}$ = 0.53, *p* = 0.47, Figure S1B). The number of individuals sampled in each species varied from 3 to 1200 and the average depth of sequencing per individual varied from 0.028 to 79.7 (Figure S2). Variation in the depth of sequencing between individuals, quantified as the coefficient of variation in base pairs sequenced, varied about 50-fold from 0.030 to 1.6 (Figure S2). There were no missing values for any of the variables investigated in this study, but there were three species with zero variant sites called (*Capsicum annuum*, *Heliosperma pusillum*, and *Papaver somniferum*) because they had very low coverage sequencing datasets (average coverages per individual of 0.035×, 0.169×, and 0.043×, respectively, Table S1). We omitted these species from all downstream analyses.

Before testing our central hypothesis, we investigated whether technical sequencing variables could explain any of the diversity values observed in our dataset. Mean coverage correlated with both nucleotide diversity (ρ=0.33,p=.00033, Figure S3A) and k-mer diversity (Jaccard: $\rho =-0.53,p=2.6\times 10^{-9}$, Figure S3D; Bray-Curtis: ρ=−0.34,p=.00021, Figure S3G). Coefficient of variation in bp sequenced correlated strongly with k-mer diversity (Jaccard: ρ = 0.36, *p* = .00013, Figure S3E; Bray-Curtis: $\rho =0.42,p=4.7\times 10^{-6}$, Figure S3H) but not nucleotide diversity (ρ=−0.088,p=.36, Figure S3B). The number of individuals sequenced did not correlate with either nucleotide diversity or k-mer diversity (Figure S3C, F, and I).

**Figure 2:**
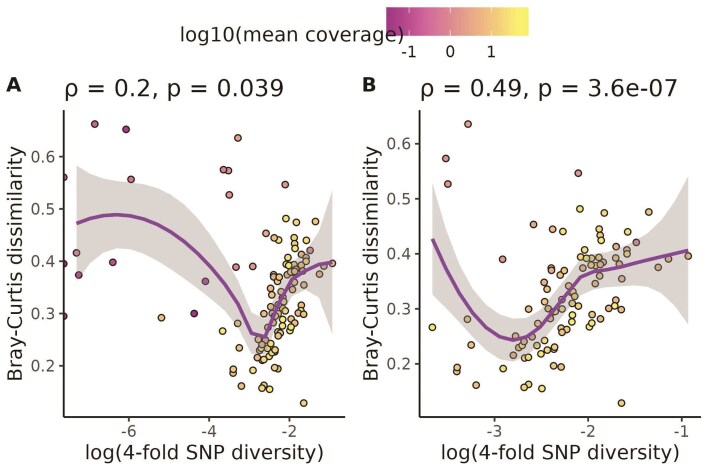
**Low coverage and low numbers of variant calls explains species with abnormally low diversity.** (A) The relationship between k-mer diversity and nucleotide diversity without omitting species with ≤ 0.5× coverage or ≤ 1,000 SNP calls. (B) The same relationship, except species with ≤ 0.5× coverage or ≤ 1,000 SNP calls are omitted. Each data point is a species. All species’ points are colored by the log (base 10) of average genome-wide coverage per individual for that species. Purple lines are loess smoothing lines with 95% confidence intervals shaded in gray. Values across the top of each plot are Spearman correlation coefficients (ρ) and *p*-values that test whether each correlation coefficient differs from zero.

Given that mean coverage strongly correlated with both k-mer dissimilarity and nucleotide diversity, we decided to investigate this relationship further. We would expect that low coverage sequencing would produce artificially low nucleotide diversity and artificially high k-mer diversity. This is because our SNP-calling pipeline is generally tuned for high-coverage datasets (see **Supplementary material**), so low coverage datasets produce few confident SNP calls. Conversely, low coverage sequencing only samples a small proportion of the total k-mer space in a set of reads. Thus, two independent low coverage samples are unlikely to share many k-mers in common and will have inflated k-mer diversity. Overall, we observed that species with very low mean coverage had both low nucleotide and high k-mer diversity (Figures 2A and S4A). In contrast, there was no clear mapping of these abnormal diversity values with coefficient of variation in base pairs sequenced (Figure S5) or the number of individuals sequenced (Figure S6). Based on these results, we removed 10 species from our dataset with mean coverage per individual ≤ 0.5×. This included three species (*C. annuum*, *H. pusillum*, and *P. somniferum*) with zero variant sites called because they had very low coverage (0.035×, 0.169×, and 0.043×, respectively, Table S1). We also dropped 4 additional species with fewer than 1,000 variant sites called: *Camelina sativa *, *Sesamum indicum*, *Setaria italica*, *Actinidia chinensis*, the first three of which were just slightly above our coverage cutoff (0.63×, 0.92×, and 0.63×, respecitively, Table S1). These filtering steps removed many of the species with abnormally low diversity and high dissimilarity values (Figure 2B). In total, we kept data for 98 species for downstream hypothesis testing.

### Range size-squared height ratio varies over more orders of magnitude than nucleotide diversity

We next investigated whether Lewontin’s paradox applied to our dataset by comparing diversity estimates against population size proxies. For each species, we estimated range size using either occurrence data from the Global Biodiversity Information Facility (GBIF, GBIF (2025), https://www.gbif.org/) occurrence data or World Checklist of Vascular Plants (WCVP, POWO (2025), https://powo.science.kew.org/) range maps. Estimates from these two methods were significantly correlated no matter whether invaded ranges (as defined in the WCVP range maps) were included (ρ = 0.31, *p* = .00096, Figure S7A) or excluded (ρ = 0.48, *p* = 7.3$\times 10^{-8}$, Figure S7B). The omission of invaded ranges lowered the range size of several plant species based on WCVP range maps (Figure S7C) but had less effect on ranges estimated from GBIF occurrence data (Figure S7D).

We then calculated the ratio of range size to squared plant height (Supplemental Equation 7) using height values from the Encyclopedia of Life (EOL, Parr et al. (2014), https://www.eol.org/) and TRY databases (Kattge et al. (2011), https://www.try-db.org/). We used this ratio as our primary population size proxy in downstream analyses. After excluding species with <0.5× coverage and <1,000 variant sites called (Figure 2), nucleotide diversity varied over about 4 orders of magnitude for the species in our dataset (from 0.00021 to 0.117, Table S2), while the ratio of range size to squared plant height based on WCVP and GBIF range estimation methods (including both native and invaded ranges) varied over 10 (from $8.9\times 10^{8}$ to $1.7\times 10^{18}$) and 13 (from $8.6\times 10^{5}$ to $1.5\times 10^{18}$) orders of magnitude, respectively (Table S2). Mean pairwise Bray-Curtis dissimilarity values varied about 4.9-fold across species, from 0.13 to 0.64, while mean pairwise Jaccard dissimilarity varied about 22-fold, from 0.040 to 0.87 (Table S2). Bray-Curtis dissimilarity values correlated with Jaccard dissimilarity values across species (ρ=0.76, *p*  $<2.2\times 10^{-16}$, Figure S8).

### k -mer diversity scales with population size proxies more than nucleotide diversity

The core of Lewontin’s paradox is that a population’s diversity does not scale much with population size. If k-mers capture a wider range of genetic variation compared with SNPs, population size will scale more with k-mer diversity than nucleotide diversity. If we did not control for shared evolutionary history or any confounding variables (mating system, life cycle habit, cultivation status, or genome size), then none of our diversity measures significantly correlated with the range size-squared height ratio (Figure S9). After controlling for confounding variables, nucleotide diversity marginally scaled with the range size-squared height ratio (β=0.14, SE = 0.056, *p* = .017, Figure S10A). However, the relationship between k-mer diversity and the range size-squared height ratio was highly significant, with generally a greater slope (Jaccard: β= 0.64, SE = 0.096, *p* = $2.2\times 10^{-9}$, Figure S10B; Bray-Curtis dissimilarity: β=0.79, SE = 0.11, *p* = $7.3\times 10^{-11}$, Figure S10C). We observed the same qualitative trend when we included both native and invaded ranges in the range size-squared height ratio (Figure S10D–F, Table S3), or used the GBIF-based range estimates instead of WCVP-based estimates (Figure S11, Table S3). Interestingly, we often observed Bray-Curtis dissimilarity having a larger slope with the range size-squared height ratio compared with Jaccard dissimilarity (β=0.64 vs. 0.79, Figure S10B and C), but models where Bray-Curtis dissimilarity was the response variable generally had lower adjusted $R^{2}$ (e.g., $R^{2}$ = 0.51 vs. 0.41, models 2 and 3 in Table S4).

We also analyzed range size and plant height separately as population size proxies (Figures S12–S14). Overall, WCVP-estimated range size significantly affected nucleotide diversity (β=0.29, SE = 0.072, *p* = .00011, Figure S12A) and k-mer diversity (Jaccard: β=0.92, SE = 0.13, *p* = 9.9$\times 10^{-11}$, Figure S12B; Bray-Curtis: β=1.2, SE = 0.13, *p* = 3.2$\times 10^{-14}$, Figure S12C), and this trend held when we estimated range size from GBIF occurrences (Figure S13A–C) or included invaded range area (Figures S12D–F and S13D–F). On the other hand, nucleotide diversity did not scale with plant height (β = 0.13, SE = 0.19, *p* = .5, Figure S14A), but k-mer diversity marginally scaled downward with plant height (Jaccard: β=−0.78, SE =0.38, *p* = .046, Figure 14B; Bray-Curtis: β=−0.77, SE = 0.44, *p* = .088, Figure S14C).

**Figure 3:**
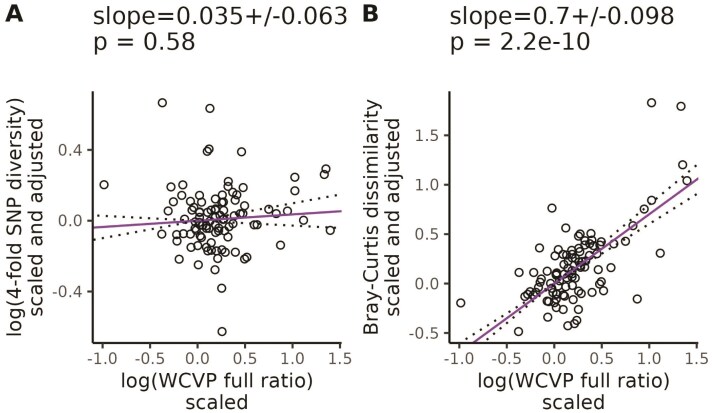
**
k -mer diversity scales with population size proxies after controlling for genome size, life cycle habit, mating system, and cultivation status.** Purple lines are partial phylogenetic regression lines between diversity levels and the population size proxy. The *y*-axis (diversity) is scaled and adjusted according to Equation 6: scaling diversity levels to a standard normal distribution, followed by correcting for phylogenetic relatedness and adjusting for the confounding variables (genome size, life cycle habit, mating system, and cultivation status). The values at the top of each plot give the slope of the partial regression ± one standard error and *p*-values testing whether the slopes differ from zero. Dotted lines show the partial regression slope ± one standard error. WCVP full ratio is a population size proxy estimated as the ratio of range size recorded in WCVP range maps (including invaded ranges) to squared plant height.

Finally, we repeated our partial phylogenetic regressions controlling for genome size as an additional covariate. In this case, nucleotide diversity did not scale with the range size-squared height ratio (β = 0.035, SE = 0.063, *p* = .58, Figure 3A), but k-mer diversity did (Jaccard: β=0.54, SE = 0.093, *p* = $8.8\times 10^{-8}$, Figure S15; Bray-Curtis: β = 0.7, SE = 0.098, *p* = 2.2$\times 10^{-10}$, Figure 3B). Again, we got qualitatively similar results when we excluded invaded ranges in our range size estimates (Figure S16), used GBIF occurrences to estimate range size-squared height ratio (Figure S17), or used WCVP range size as the population size proxy (Figure S18). However, GBIF range size by itself did not scale with Jaccard dissimilarity (Figure S19B and E). Increased plant height associated with decreased k-mer diversity but had no significant relationship with nucleotide diversity (Figure S20).

### k -mer diversity scales with genome size more than nucleotide diversity

We also investigated the relationship between diversity and genome size because we expected genome size to potentially play a role in the mechanism underlying the greater scaling of k-mer diversity with population size. Genome size is often a strong predictor of diversity (Lynch and Conery, 2003). Among eukaryotes, variation in genome size is largely explained by variation in transposable element abundance (Flavell et al., 1974; Kidwell, 2002; Lynch and Conery, 2003; Muñoz-Diez et al., 2012; Tenaillon et al., 2011; Nystedt et al., 2013; Ibarra-Laclette et al., 2013), which contribute substantially to the repetitive sequence content of genomes and increase the difficulty of aligning short reads to a reference genome (reviewed in Goerner-Potvin and Bourque (2018)). Thus, our expectation was that k-mer-based diversity measures are more sensitive to genome size variation compared with nucleotide diversity.

**Figure 4:**
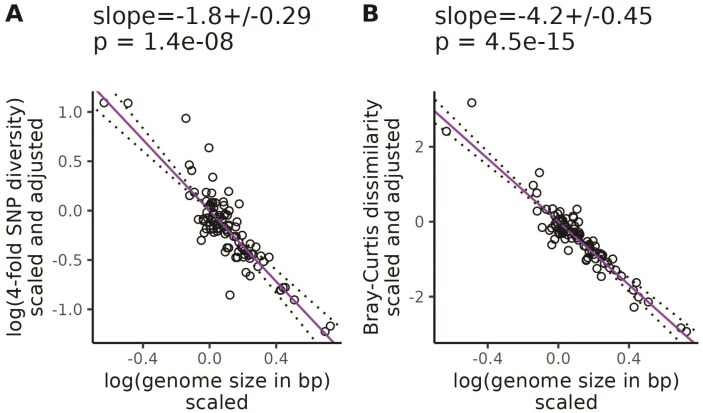
**
k -mer diversity is more sensitive to genome size variation than nucleotide diversity.** Purple lines are partial phylogenetic regression lines between diversity levels and genome size (see Equation 6) after scaling diversity levels to a standard normal distribution (mean = 0, variance = 1), followed by scaling diversity levels and population sizes according to their phylogenetic relatedness, and finally adjusting for the confounding effects of mating system, cultivation status, life cycle habit, and population size. Here, we used the ratio of range size to squared plant height, where range size was estimated from ranges in WCVP range maps (including invaded ranges). The values at the top of each plot give the slope of the partial regression ± one standard error and *p*-values testing whether the slopes differ from zero. Dotted lines show the partial regression slope ± one standard error.

Increasing genome size was associated with decreasing k-mer diversity (Jaccard: β=−3.7, SE = 0.42, *p* = 8.4$\times 10^{-14}$, Figure S21; Bray-Curtis: β=−4.2, SE = 0.45, *p* = 4.5$\times 10^{-15}$, Figure 4B) and nucleotide diversity (β−1.8, SE = 0.29, *p* = $1.4\times 10^{-8}$, Figure 4A), after controlling for variation in the range size-squared height ratio, mating system, life cycle habit, cultivation status, and evolutionary history. We got qualitatively similar results when the population size proxy we corrected for excluded invaded ranges (Figure S22), or if our population size proxy was based on GBIF occurrences (Figure S23), or we used range size or plant height individually to control for population size variation (Figures S24–S26). Across all of these analyses, the partial regression relationship between genome size and diversity was always significantly negative.

## Discussion

Our goal was to investigate whether genomic approaches that can capture more genetic variation than reference-based methods can improve the scaling between population size proxies and diversity. This was motivated by literature suggesting suggesting SNPs called against a single reference provide an incomplete picture of genome-wide polymorphism (Schmidt et al., 2021; VanWallendael and Alvarez, 2022; Jaegle et al., 2023; Sopniewski and Catullo, 2024). In total, we processed >205 terabases of publicly available sequencing data from the SRA over ∼12 months of wall time, split between a maximum of 512 cores and 50 TB of disk space. After carefully accounting for potential technical and phylogenetic confounding, the standardized slope between k-mer-based diversity and the range size-squared height ratio was up to 20 times larger than the same standardized slope for nucleotide diversity (β= 0.035 vs. 0.7, Figure 3). We observed similar results across the two different measures of range size (Figure S17) and k-mer diversity (Figure S15). We also observed that k-mer-based diversity is more sensitive to variation in genome size compared with nucleotide diversity (Figure 4). Overall, these results are consistent with the possibility that diversity missed by reference-based analyses or changes in the diversity calculation itself can partly explain the weak scaling between population size and diversity. However, a larger pangenome scale analysis will be required to fully understand the genetic variants underlying this result.

Extending our findings to Lewontin’s paradox directly is complicated by a few factors. First, it would be ideal to compare our diversity estimates to neutral expectations of diversity vs. population size. For SNPs, the expected diversity under neutrality would be $4N_{e}\mu$ or a similar formula. For k-mers, the exact value of μ is not clear, as k-mers also reflect non-SNP variation. Several k-mer-based measures of diversity are nearly perfectly correlated with π in neutral models where only SNPs are considered (Roberts et al., 2024), but the inclusion of non-SNP variation would probably change this relationship. Identifying the concrete variants underlying k-mers would help us better understand μ for k-mer, but this is currently not possible for mutations other than SNPs and small indels without pangenomic references (Uricaru et al., 2015; Gauthier et al., 2020). The eventual release of pangenomic references across many species will provide better context for increased scaling of k-mer diversity with population size. Similar to previous Lewontin’s paradox studies, we also use both noncoding sequences (for k-mers) and fourfold degenerate sites (for SNPs) as standards for estimating neutral diversity (Leffler et al., 2012; Buffalo, 2021), but there could be differences between coding and noncoding values of μ that contribute to results observed in our work and previous work. More estimates of μ are needed for non-SNP mutations (Quiroz et al., 2023), but we further caution that many estimates of μ will be limited in that they usually reflect contemporary populations. Variation in μ over time, such as through TE bursts (Belyayev, 2014), could play an important role in diversity vs. population size scaling and warrant further research.

In addition to limitations estimating μ and π, estimates of $N_{e}$ also tend to be limited in investigations of Lewontin’s paradox. Like previous analyses, our analysis assumes that contemporary population size estimates are good proxies for historic population sizes (Corbett-Detig et al., 2015; Buffalo and Coop, 2020). Common population size proxies such as range size and plant height only reflect the current census population size of a species. However, it is the long-term harmonic mean of the effective population size, not just the current size, that determines diversity levels within a population (Wright, 1940). There is some literature correlating aspects of species history with contemporary diversity levels (López-Delgado and Meirmans, 2022) and incorporating these metrics into Lewontin’s paradox is an exciting avenue for future research. As a first step in this direction, we used plant range maps into native and invaded ranges to test the robustness of our results to invasion-related range size changes (Brown et al., 2023). Overall, our observations were remarkably similar no matter whether we included or excluded invaded ranges in our population size proxies (Figure S17A–C vs. D–F). Part of this apparent robustness was due to the insensitivity of our GBIF-based range size estimates to the inclusion of invaded ranges (Figure S7D). However, our WCVP-based range size estimates were drastically altered by the inclusion of invaded ranges (Figure S7C) and still yielded similar results (Figures 3, S15, and S16). Although we cannot rule out the possibility that older historical events have affected contemporary diversity levels, our results appear to be robust to recent biological invasions.

As with all regression-based analyses, our results are also ultimately sensitive to error in the measurement of both covariates (population size proxies, genome size, mating system, life cycle habit, or cultivation status) and outcome variables (nucleotide or k-mer diversity). For example, we did not filter out potentially contaminating sequences from our k-mer analysis, which could add noise to our k-mer dissimilarity values. However, this could only explain the increased scaling between k-mer diversity and population size if plants with larger population size proxies had a higher diversity of contaminants, which seems unlikely given that many of the sequencing datasets in our study are from laboratory cultivated plants (see Table S1) and that we excluded low-frequency k-mers from our analyses. Our study is also unique in the multiple steps we took to limit the influence of systematic measurement errors on our coefficients, including reanalyzing all population-level sequencing data with one pipeline to limit the impact of bioinformatic parameter choices on our analysis (Mirchandani et al., 2024), filtering out low coverage datasets(Sandell et al., 2022), accounting for missing data in calculations of nucleotide diversity (Schmidt et al., 2021; Korunes and Samuk, 2021), and estimating range size with two different methods (WCVP range maps and GBIF occurrence records, Figure S6). Although we could not control for some covariates (Willis, 1922; Romiguier et al., 2014; Guo et al., 2024) due to a dearth of data, our study is still the largest reanalysis of population-level sequencing data in plants that we know of to date. The availability of our workflow also makes it easy for our study to be extended as more population-level sequencing data is released.

Interestingly, the estimated effect of our population size proxies on diversity was often slightly larger for Bray-Curtis dissimilarity than Jaccard dissimilarity (for example, β=0.7 vs. 0.54 from Figure 3B vs. Figure S15, Table S4). In contrast, the range size-squared height ratio was often slightly more predictive of Jaccard dissimilarity than Bray-Curtis dissimilarity (Table S4). We could not test whether these trends were statistically significant, but the benefits of different k-mer metrics in predicting measures of population size warrant further study. Our expectation is that k-mer diversity measures based on frequency, such as Bray-Curtis dissimilarity, better capture diversity compared with measures based on purely k-mer presence/absence, such as Jaccard dissimilarity, because they explicitly measure copy number variation. However, accurately measuring k-mer frequencies likely requires higher sequencing coverage than calling presence/absence, which could explain why Bray-Curtis dissimilarity generally scaled more with population size but had a lower $R^{2}$ compared with Jaccard dissimilarity (Table S4). Future studies using higher coverage population level sequencing data could help test this hypothesis.

k -mer frequencies are known to be highly informative of genomic structure, with one common application of k-mers being the estimation of genome size (Vurture et al., 2017; Pflug et al., 2020). Similar to previous studies, we observed that nucleotide diversity was negatively correlated with genome size (Lynch and Conery, 2003; Chen et al., 2017), but we observed an even stronger negative correlation for k-mer diversity (β=−1.8, SE = 0.29 vs. β= -3.7, SE = 0.42 in Figure 4). k-mers also appeared to explain diversity patterns that scaled with population size beyond those explained by genome size, while nucleotide diversity did not. After controlling for genome size, the relationship between our population size proxies and nucleotide diversity was not significant (Figure 3A, S17–S19 panels A and D), but the relationship between k-mer diversity and population size proxies was often still highly significant (Figure 3B, S17–S19 panels B; C; E; F). The only exception was that Jaccard dissimilarity did not significantly scale with GBIF-based estimates of range size (Figure S19B and E). This additional scaling of k-mer diversity with population size beyond just the effects of genome size and confounding variables suggests that k-mers capture some element of the population size-diversity relationship that is absent from nucleotide diversity.

Our results do not negate the fact that other important factors also underlie Lewontin’s paradox, such as past demographic fluctuations and linked selection. However, our results do suggest that future studies of Lewontin’s paradox may want to consider diversity outside one reference genome. The increasing availability of pangenomes across species (Göktay et al., 2021; Zhou et al., 2022; Rice et al., 2023; Wang et al., 2023) offers many opportunities to revisit this classic population genetics question. Ideal future studies would use pangenomic genotyping methods across a wide range of species with a standardized pipeline, combined with multiple proxies of population size, and understandings of each species’ demographic history. Altogether, these methodological developments will hopefully reveal a more wholistic picture of variation across the tree of life.

## Supplementary Material

qraf011_suppl_Supplementary

qraf011_suppl_Supplementary_Tables_S1

qraf011_suppl_Supplementary_Tables_S2

qraf011_suppl_Supplementary_Tables_S3

qraf011_suppl_Supplementary_Tables_S4

## Data Availability

The code for our entire analysis is packaged as a snakemake workflow stored here: https://github.com/milesroberts-123/tajimasDacrossSpecies. Our Matrices of k-mer counts, VCF files of filtered SNP calls, the dataframe of covariates we used for statistical analyses (including population size proxies), and the phylogenetic tree we used for statistical analyses are all available at Dryad: https://doi.org/10.5061/dryad.s1rn8pkk0.
