# Supplementary material for: k-mer-based diversity scales with population size proxies more than nucleotide diversity in a meta-analysis of 98 plant species
Source: Evol Lett. 2025 Jun 12;9(4):434–45. doi: 10.1093/evlett/qraf011 (PMC12448231; doi:10.1093/evlett/qraf011)
Supplement: qraf011_suppl_Supplementary [file qraf011_suppl_supplementary.pdf]

Supplemental material for: *k-mer-based diversity scales with population size proxies more than nucleotide diversity in a meta-analysis of 98 plant species*

Miles Roberts and Emily Josephs

2025-03-27

Contents

1 Supplemental methods **2**
1.1 Population-level sequencing data collection . . . 2
1.2 SNP calling pipeline . . . 2
1.3 *k*-mer counting pipeline . . . 4
1.4 Population size proxies . . . 5
1.4.1 Range size estimation from GBIF occurrence data . . . 5
1.4.2 Range size estimation from WCVP distribution maps . . . 7
1.4.3 Population density estimation from plant height . . . 7
1.5 Labeling species with genome size, mating system, ploidy, cultivation status, and life cycle habit 7

2 Supplemental figures: Exploring relationships in data before outlier removal **13**

3 Supplemental figures: Population size proxy vs diversity relationships after controlling for phylogeny and life-history variables, but not controlling for genome size **22**

4 Supplemental figures: Population size proxy vs diversity relationships after controlling for phylogeny, life-history variables, and genome size **27**

5 Supplemental figures: Genome size vs diversity relationships after controlling for population size proxies, phylogeny, and life-history variables **33**

6 Supplemental Table captions **39**

1 Supplemental methods

1.1 Population-level sequencing data collection

We started by building a list of species with high quality, publicly available reference genomes as well as population-level sequencing data. The source for the genome assembly and annotation used for each species in this study is listed in Table S1. We first downloaded all genomes in Phytozome (https://phytozome-next.jgi.doe.gov/) with unrestricted data usage. We then downloaded all genomes for species from Ensembl plants (https://plants.ensembl.org/index.html) that were not already represented in Phytozome. Next, we downloaded genomes for additional species from the NCBI genome database (https://www.ncbi.nlm.nih.gov/genome/) that were not already present in either Phytozome or Ensembl and met all of the following criteria:

- matched filters: eukaryotic, plants, land plants, and exclude partial
- included assemblies of nuclear DNA (i.e. not just plastid genomes)
- included annotations of coding sequences

We also downloaded a genome for *Nicotiana tabaccum* from the Sol genomics network (https://solgenomics.net/). Finally, we omitted 8 species (*Aegilops tauschii*, *Hordeum vulgare*, *Lens culinaris*, *Pisum sativum*, *Thinopyrum intermedium*, *Trifolium pratense*, *Triticum aestivum*, *Triticum turgidum*) that had at least one chromosome longer than 2^{29} bp (about 537 Mb) from all downstream analyses because tabix indexing, which is often utilized for SNP-calling pipelines, does not support chromosomes exceeding this length. In the end, we were left with genome assemblies and annotations for 112 plant species (see Table S1).

Note that, similar to previous studies (Corbett-Detig et al., 2015; Buffalo, 2021), many of the plant species in this set of 112 are domesticated (see Table S1). This means that many of the species in our dataset have likely undergone recent demographic changes. However correctly accounting for demography is a general limitation of Lewontin's paradox studies, as we can only estimate proxies of N_e for contemporary populations (Corbett-Detig et al., 2015; Buffalo, 2021). We include cultivation status in our downstream modeling to help account for systematic differences between cultivated and wild species (see **Statistical analysis**) and also repeat our analysis with multiple types of population size proxies.

For each species with a reference genome, we searched for DNA-seq runs in the National Center for Biotechnology Information's Sequence Read Archive (SRA) with a name in the organism field that matched the species name (e.g. search for Arabidopsis lyrata[Organism] to get *Arabidopsis lyrata* runs). We downloaded the run info for each search and found the study with most sequenced individuals for inclusion in our analysis. Most datasets came from individual studies, with the exception of *Zea mays*, which included several studies described in Bukowski et al. (2017). The datasets used for each species are listed in Table S1.

We limited the size of each species' dataset to no more than 7.5×10^{12} bp and no more than 1200 individuals because this defined the amount of data our workflow could process without the peak memory limit exceeding 50 TB and the time limit for genotype calling exceeding 7 days. If a species' dataset exceeded either 1200 individuals or 7.5×10^{12} bp, we randomly dropped one individual at a time until both of these limits were satisfied.

We downloaded the SRA runs associated with each individual using the SRA toolkit (v2.10.7), then trimmed low-quality base calls with fastp (v0.23.1, Chen et al. (2018)), requiring a minimum quality score of 20 and a minimum read length of 30 base pairs. For each species, we summarized the results of fastp trimming using multiqc (v1.18, Ewels et al. (2016)). After trimming, any fastq files that were technical replicates of the same individual were concatenated. Concatenated fastq files were then processed through two different workflows: SNP-calling and k-mer counting.

1.2 SNP calling pipeline

We aligned sequencing reads for each individual to their respective reference genome using BWA MEM (v0.7.17, Li and Durbin (2009); Li (2013)), sorted the resulting BAM files with samtools (v1.11, Danecek et al. (2021)), and marked optical duplicates with picardtools (picard-slim v2.22.1, Institute (2019)). Next,

we called SNPs with GATK HaplotypeCaller (v4.1.4.1, McKenna et al. (2010); Poplin et al. (2018)). We varied the –ploidy parameter for HaplotypeCaller between species depending on the actual ploidy recorded in the literature and whether individual subgenome assemblies were available. However, the vast majority of species in our dataset had a –ploidy paramter of 2. We restricted genotype calling to only 4-fold degenerate sites within the nuclear genome and included all nuclear genome scaffolds, as identified by degenotate (v1.1.3, Mirchandani et al. (2024)), to focus solely on neutral diversity. Notably, we chose to not call SNPs in non-coding sequences in plants because plant genomes tend to be highly repetitive and are largely composed of transposable elements, making short read mapping difficult For example, even in the small-genome plant species *Capsella grandiflora*, SNPs can only be confidently called in 10% of intergenic sites (Williamson et al., 2014). Runs for each species were then combined with GATK GenomicsDBImport, then genotyped with GATK GenotypeGVCFs, including invariant sites as done in Korunes and Samuk (2021). Variant and invariant sites were separated with bcftools (v1.17, Danecek et al. (2021)) and then filtered separately, as recommended by Korunes and Samuk (2021). Variant sites were removed from our analyses if they met at least one of the following criteria: number of alleles > 2, indel status = TRUE, fraction of missing genotypes > 0.2, QD < 2.0, QUAL < 30.0, MQ < 40.00, FS > 60.0, HaplotypeScore > 13.0, MQRankSum < -12.5, and ReadPosRankSum < -8.0. In short, these filters reflect the standard population genetics practices of removing non-biallelic SNPs and SNPs with a lot of missing genotype calls. The exact filter cutoffs also come from GATK Best Practices for hard filtering (Caetano-Anolles, 2023) that is widely used (e.g. (Yu et al., 2024; Ramirez-Ramirez et al., 2024; Glasenapp and Pogson, 2024; Ritter et al., 2024)). For each species, we also required that each variant site have a minimum read depth of 5, but no more than 3 times the genome-wide average read depth at variant sites for that species. We chose a minimum read depth of 5 to maintain comparability with our k-mer dissimilarity calculations which required minimum k-mer counts of 5 (see **k-mer counting**), but we also filtered out calls with very high read depths because these could represent read mismapping due to repetitive or paralogous regions. Meanwhile, invariant sites were removed from our analyses if they met at least one of the following criteria: QUAL > 100.0, read depth ≤ 5, or read depth ≥ 3 times the genome-wide average read depth at invariant sites for that species. Finally, invariant and variant sites were concatenated into a single VCF file per scaffold using bcftools. For *Brassica napus* and *Miscanthus sinensis*, scaffolds named "LK032656" (195,249 bp, 0.023 % of the genome) and "scaffold04645" (2,838 bp, 0.000136 % of the genome), respectively, were omitted from our analyses because an error in SLURM job cancellation caused snakemake to prematurely delete intermediate files for these scaffolds. It is worth noting that different choices of genotype callers and filtering parameters could lead to different estimates of nucleotide diversity. However, our workflow is representative of SNP calling workflows used in many published population genetic analyses.

Using the SNP genotypes called from our pipeline, we then calculated genome-wide average nucleotide diversity at four-fold degenerate sites ($\bar{\pi}$) using the filtered set of variant and invariant sites. To do this, we first calculated heterozygosity at each four-fold degenerate site (i) according to Hahn (2018):

$$\pi_i = \left(\frac{n_i}{n_i - 1}\right)\left(1 - \sum_{j=1}^{a_i} p_{ij}^2\right) \tag{1}$$

where n_i is the number of sequenced chromosomes with non-missing genotypes for site i, a_i is the number of alleles for site i, and p_{ij} is the frequency of the jth allele at site i. For each invariant site, the equation reduces to $\pi_i = 0$ because $p_{i1} = 1$ and $a_i = 1$. To get $\bar{\pi}$, we then calculated the average value of π_i across all M sites in the genome (including both variant and invariant sites):

$$\bar{\pi} = \frac{\sum_{i=1}^{M} \pi_i}{M} \tag{2}$$

Importantly, this calculation of nucleotide diversity does not assume that all missing genotype calls are homozygous for the reference genotype, meaning that our estimates of π will not be downwardly biased by missing data (Korunes and Samuk, 2021).

1.3 k-mer counting pipeline

For each species, we counted 30-mers in the coding sequences of their respective reference genome using KMC (v3.2.1, Kokot et al. (2017)). We chose to count k-mers of 30 base pairs (i.e. 30-mers) for all species in our dataset because previous k-mer-based analyses in plants typically analyzed k-mers in the range of 20 - 40 base pairs (Voichek and Weigel, 2020; Kim et al., 2020; VanWallendael and Alvarez, 2022; Ruperao et al., 2023) and because k-mers in this range can be reliably sequenced with short reads while capturing the majority of unique genomic sequences (Shajii et al., 2016; Ondov et al., 2016; Roberts et al., 2024). Next, we removed any 30-mers that matched 30-mers found in each species' corresponding set of coding sequences. This step intended to focus our k-mers down to a set that is evolving more neutrally on average, analogously to how we focused on only 4-fold degenerate SNPs in our SNP-calling pipeline. The justification for this approach is that non-coding sequences generally have weaker signals of interspecies conservation compared to coding sequences (Woolfe et al., 2005; Siepel et al., 2005; Johnsson et al., 2014). Although, similarly to 4-fold degenerate sites, many studies have observed non-coding sequences under selective constraints (Margulies et al., 2003; Guo et al., 2007). Thus, similar to the common analysis of 4-fold degenerate sites, our k-mer analysis is limited by an inability to completely remove the effects of selection on sequence diversity.

Although comparing our k-mer and nucleotide diversity metrics will be affected by differences between coding and non-coding sequences, many previous studies found that non-coding regions and 4-fold degenerate sites have very similar levels of diversity (Moriyama and Powell, 1996; Makałowski and Boguski, 1998; Halushka et al., 1999; Zwick et al., 2000; Tenaillon et al., 2001; Nordborg et al., 2005; Branca et al., 2011; Williamson et al., 2014; Wang et al., 2016; Phung et al., 2016; Mattila et al., 2017). Previous investigations of Lewontin's paradox also found that diversity levels across species vary much more than diversity levels across different categories of putatively neutral sequences (Leffler et al., 2012; Buffalo, 2021) and subsequently pooled estimates of neutral diversity across different categories of sites. Our analysis choices here are thus in line with previous studies; however, larger scale pangenomic analyses will be helpful in relaxing this assumption.

For most species in this study, we identified hundreds of millions of unique 30-mers. It would be computationally expensive to analyze all the 30-mers for every species. However, previous studies have shown that one can randomly downsample k-mer sets with very minimal effects on measures of genomic dissimilarity (Fofanov et al., 2004; Benoit et al., 2020; Roberts et al., 2024). Thus, we randomly downsampled each species' 30-mer list to 10 million 30-mers with a frequency ≥ 5 in at least one sample in the species' 30-mer list. We chose to downsample to 10 million 30-mers to decrease disk space burden of storing k-mer counts and because several previous studies show that subsets of only 1 million k-mers or less can reliably estimate genetic dissimilarity in many systems (Ondov et al., 2016; Benoit et al., 2020; VanWallendael and Alvarez, 2022). The reason we also included a frequency cut-off of 5 is to omit k-mers containing sequencing errors, which predominantly occur as very low-frequency k-mers (Ranallo-Benavidez et al., 2020). We chose a frequency cutoff of 5 to to include more k-mers in some of our lower coverage datasets, but frequency cut-offs anywhere from 2-10 are used in plants (Voichek and Weigel, 2020; VanWallendael and Alvarez, 2022). We then joined the subset k-mer counts for each individual into a single matrix for each species. We used this k-mer frequency matrix to measure genetic distance in two ways. First, we calculated Jaccard dissimilarity (J_D, Ondov et al. (2016)) between each pair of individuals in a species' dataset as:

$$J_D(X,Y) = 1 - \frac{X \cap Y}{X \cup Y} \tag{3}$$

where X and Y represent sets of unique k-mers identified as present in two different read sets. A k-mer is defined as present if it's frequency in a sample is ≥ 5. To get the genome-wide average Jaccard dissimilarity ($\bar{J_D}$), we took the average of all the pairwise Jaccard dissimilarities.

Jaccard dissimilarity is likely the most commonly used k-mer-based diversity measure (Ondov et al., 2016). However, whether a k-mer reaches the frequency threshold needed to be identified as preset in a sample depends on the sequencing depth for the sample (VanWallendael and Alvarez, 2022). Thus, we also calculated Bray-Curtis dissimilarity (B_D) between each pair of individuals in a species' dataset as:

$$B_D(X,Y) = 1 - \frac{2\sum_i^k \min(m_i^*(X), m_i^*(Y))}{\sum_i^k m_i^*(X) + m_i^*(Y)} \tag{4}$$

where $m_i^*(X)$ gives the normalized frequency of k-mer i in genome X. The normalized frequencies are calculated by taking each frequency $m_i(X)$ and dividing it by the sum of the raw frequencies as in Dubinkina et al. (2016):

$$m_i^*(X) = \frac{m_i(X)}{\sum_i m_i(X)} \tag{5}$$

This step accounts for variation in coverage between samples on k-mer frequency. To get the genome-wide average Bray-Curtis dissimilarity ($\bar{B_D}$), we again took the average of all the pairwise Bray-Curtis dissimilarities. Note that both Jaccard and Bray-Curtis dissimilarity are scaled in their denominators by either the total number of unique k-mers or total number of k-mers respectively, analogous to how nucleotide diversity is scaled by the number of sites included in the calculation.

1.4 Population size proxies

Following similar methods to Corbett-Detig et al. (2015) and Buffalo (2021), we defined current census population size (N) as the product of species range size (R) in square kilometers and population density (D) in individuals per square kilometer:

$$N = RD \tag{6}$$

Estimation of both R and D are handled separately below. Importantly, these methods have the same drawback as described in Corbett-Detig et al. (2015) and Buffalo (2021): contemporary estimates of R and D do not necessarily reflect the historical values of R and D. However, since nearly all the species in this study lack long-term historical data on their population size, it is not currently possible to estimate long-term historical N without making strong assumptions.

1.4.1 Range size estimation from GBIF occurrence data

We first estimated range size based on Global Biodiversity Information Facility (GBIF) occurrence data from the rgbif package (Chamberlain and Boettiger, 2017). For each species, we identified its GBIF taxon key(s). If the species is domesticated, we used the taxon key(s) for a wild relative with an overlapping range when possible. We then downloaded all records associated with each taxon key that had an occurrence status of "PRESENT", had coordinates that mapped to land, had any basis of record other than "FOSSIL SPECIMEN", and recorded anywhere in a year $\geq$ 1943 and $\leq$ 2023. In addition, the records could not have any GBIF issue codes, except the following issue codes were allowed:

- "AMBIGUOUS_COLLECTION"
- "AMBIGUOUS_INSTITUTION"
- "COLLECTION_MATCH_FUZZY"
- "COLLECTION_MATCH_NONE"
- "CONTINENT_DERIVED_FROM_COORDINATES"
- "COORDINATE_ROUNDED"
- "COUNTRY_DERIVED_FROM_COORDINATES"
- "COUNTRY_MISMATCH"

- "DEPTH_MIN_MAX_SWAPPED"
- "DEPTH_NON_NUMERIC"
- "DEPTH_NOT_METRIC"
- "DEPTH_UNLIKELY"
- "DIFFERENT_OWNER_INSTITUTION"
- "ELEVATION_MIN_MAX_SWAPPED"
- "ELEVATION_NON_NUMERIC"
- "ELEVATION_NOT_METRIC"
- "ELEVATION_UNLIKELY"
- "GEODETIC_DATUM_ASSUMED_WGS84"
- "INSTITUTION_COLLECTION_MISMATCH"
- "INSTITUTION_MATCH_FUZZY"
- "INSTITUTION_MATCH_NONE"
- "OCCURRENCE_STATUS_INFERRED_FROM_BASIS_OF_RECORD"
- "OCCURRENCE_STATUS_INFERRED_FROM_INDIVIDUAL_COUNT"

Similar to previous studies (Corbett-Detig et al., 2015; Buffalo, 2021), we estimated range size for domesticated species using GBIF occurrences from closely-related wild relatives because it is difficult to distinguish the native and introduced ranges of globally cultivated crop species with only occurrence data. Note, however, that we also used an additional method for estimating range size that is not burdened by this same assumption (see **Range size estimation from WCVP distribution maps**). The relatives used for each domesticated species is detailed in Table S1.

We followed methods of Buffalo (2021) to estimate range size from each species' set of GBIF occurrence data using the package alphahull (Pateiro-Lopez and Rodriguez-Casal, 2022). We started with splitting the occurrence data by continent, in order to avoid estimating ranges that overlapped with oceans. We also only kept occurrences with unique latitude-longitude values to reduce the computational burden of alphahull's algorithms. We then added a small amount of random jitter (normally distributed with $\mu = 0$ and $\sigma = 1 \times 10^{-3}$) to the latitude-longitude coordinates of each unique occurrence to avoid errors in the triangulation algorithm of alphahull, which can break when there are lots of colinear points. Finally, we filtered out any continents which had fewer than 20 unique occurrences of a species. The only exceptions to this rule were *Solanum stenotomum*, *Dioscorea alata*, and *Rhododendron griersonianum*, for which we only required 8, 6, and 3 occurrences respectively due to the rarity of these species and thus a paucity of occurrence data. We then used alphahull to compute the alpha shape of each continent subset, which can be thought of as the smallest possible convex shape that encloses a set of points in a plane. We defined the alpha parameter for the alphahull package to be 200. We then used the R packages sf (Pebesma, 2018) and rworldmap (South, 2011) to measure the sizes of the alpha shapes in square kilometers after projecting them onto the Earth's surface. Finally, we took the estimated range polygons and filtered out ones that resided on continents in the introduced range of the species, as defined by the World Checklist of Vascular Plants (WCVP) (Govaerts et al., 2021). The sum of the areas of the remaining polygons was our estimate of range size.

1.4.2 Range size estimation from WCVP distribution maps

We also estimated range size from expert-drawn species distribution maps instead of species occurrence data. We used the rWCVP package (Brown et al., 2023) to download distribution maps from WCVP (Govaerts et al., 2021). We then estimated range size for each species as either (1) the sum of the areas of all map elements labeled as "native" or "extinct" for that species or (2) the sum of the areas of all map elements labeled as "native", "invaded", or "extinct" for that species. Regions with an occurrence label of "dubious" were excluded from downstream analyses. In contrast to GBIF-derived ranges, we used distribution maps for domesticated species in this estimate of range size because the maps discriminate between the native and introduced ranges of species.

1.4.3 Population density estimation from plant height

Similarly to previous studies, we use plant height as a proxy for plant population density (Corbett-Detig et al., 2015). While it would be ideal to use actual population densities in our analyses, we could not find published estimates of population densities for many of the species in our dataset and all previous studies investigating Lewontin's paradox rely on population size proxies (Leffler et al., 2012; Corbett-Detig et al., 2015; Filatov, 2019; Buffalo, 2021). We elaborate further on the limitations of using proxies in the Discussion, but at the time of writing this manuscript using proxies is the only way to achieve a sufficient sample size for investigating Lewontin's paradox.

We decided to use plant height rather than plant mass (Deng et al., 2012) as our measure of body size because plant height measurements are available for many more species in our dataset and also to make our results more comparable to previous studies that also use plant height (Corbett-Detig et al., 2015). According to theory outlined in Deng et al. (2012), where D is population density, M is plant mass, and h is plant height, $D \propto M^{-3/4}$ and $M \propto h^{8/3}$. Combining these two relationships gives $D \propto (h^{8/3})^{-3/4}$ which simplifies to $D \propto h^{-2}$. Adding this density-height relation to equation 6 gives our main proxy for population size:

$$N \propto \frac{R}{h^2} \tag{7}$$

In our subsequent analyses, we refer to Equation 7 as the range size-squared height ratio and we convert R to square meters and h to meters to make the ratio unitless. As Equation 7 suggests, we do not expect the range size-squared height ratio to exactly equal the true population size or be interpretable as a number of individuals. Rather, it is a quantity we expect to scale with population size. To calculate the range size-squared height ratio for each species, we downloaded plant height data from the Encyclopedia of Life (EOL, Parr et al. (2014), `https://www.eol.org/`), which mainly comprised records summarized from the TRY database (Kattge et al. (2011), `https://www.try-db.org/`). If no height measurements were available for a species in the EOL, then we used estimates we found in published scientific literature. The only exceptions to this were *Vanilla planifolia* and *Rhododendron griersonianum*, where our height estimates came from the Kew Botanical Gardens' and the American Rhododendron Society's websites, respectively. The sources used for each height value are cited in Table S1.

1.5 Labeling species with genome size, mating system, ploidy, cultivation status, and life cycle habit

For determining genome size, we used estimates from flow cytometry and k-mer-spectra analyses whenever possible instead of using assembly size, since most assemblies do not contain the entire genome of the sequenced species. Most of our genome size estimates were 1C values acquired from publications cited in the Plant DNA C-values Database (Pellicer and Leitch, 2020). Any estimates in terms of picograms (pg) of DNA were converted to base pairs using the following conversion factor: DNA in Mb = DNA in pg $\times 0.978 \times 10^9$ (Doležel et al., 2003). If genome sizes in terms of pg were not available for a species, then we used the size of the species' genome assembly as the genome size.

We next labeled each species with a mating system (selfing, outcrossing, mixed, or clonal), cultivation status (wild or cultivated), and life cycle habit (annual, biennial, perennial, or mixed) because previous studies showed these factors to be important determinants of diversity in plants (Chen et al., 2017). For classifying species into different mating systems, we used methods similar to a previous study (Opedal et al., 2023) and generally considered species with outcrossing rate < 10 % as “selfing”, species with outcrossing rate between 10 - 90 % as “mixed”, and species with outcrossing rate > 90 % as “outcrossing” when estimates of outcrossing rates were available. In the absence of outcrossing rate data, we also labeled species described as generally self-incompatible as “outcrossing” and we labeled species described as selfing as “selfing”. The only exception to this was *Oryza brachyantha* for which we could not find mating system descriptions in peer-reviewed literature. Thus, we assumed that this species was most likely outcrossing because most of the other wild *Oryza* species in the dataset were classified as outcrossing. Because of the low number of mixed (14) and clonal (2) species in our dataset, we collapsed the selfing, mixed, and clonal species into a single “not outcrossing” category for later downstream analysis. Similarly, for life cycle habit, our dataset contained only 1 biennial species and 2 species that had a mixture of annual, biennial, and perennial forms. We combined these species with the perennial category to create a single “not annual” category. For cultivation status, we looked up each species in the EOL and classified species that had documented human uses (such as for food, fiber, fodder) or had some countries known to cultivate the species as “cultivated”. All other species that did not meet these criteria were classified as “wild”. The only exception to this was *Lactuca sativa*, which did not have any human uses listed in EOL at the time of writing this paper; however, it is commonly known as lettuce so we classified it as “cultivated”. Finally, for ploidy levels, when more than one cytotype was described as present within a species we labeled the species with it’s most common naturally-occurring cytotype. Citations to relevant literature used for each classification decision can be found in Table S1.

References

Benoit, G., Mariadassou, M., Robin, S., Schbath, S., Peterlongo, P., and Lemaitre, C. (2020). SimkaMin: fast and resource frugal de novo comparative metagenomics. *Bioinformatics*, 36(4):1275–1276.

Branca, A., Paape, T. D., Zhou, P., Briskine, R., Farmer, A. D., Mudge, J., Bharti, A. K., Woodward, J. E., May, G. D., Gentzbittel, L., Ben, C., Denny, R., Sadowsky, M. J., Ronfort, J., Bataillon, T., Young, N. D., and Tiffin, P. (2011). Whole-genome nucleotide diversity, recombination, and linkage disequilibrium in the model legume *Medicago truncatula*. *Proceedings of the National Academy of Sciences*, 108(42):E864–E870. Publisher: Proceedings of the National Academy of Sciences.

Brown, M. J. M., Walker, B. E., Black, N., Govaerts, R. H. A., Ondo, I., Turner, R., and Nic Lughadha, E. (2023). rWCVP: a companion R package for the World Checklist of Vascular Plants. *New Phytologist*, 240(4):1355–1365. _eprint: https://onlinelibrary.wiley.com/doi/pdf/10.1111/nph.18919.

Buffalo, V. (2021). Quantifying the relationship between genetic diversity and population size suggests natural selection cannot explain Lewontin’s Paradox. *eLife*, 10:e67509. Publisher: eLife Sciences Publications, Ltd.

Bukowski, R., Guo, X., Lu, Y., Zou, C., He, B., Rong, Z., Wang, B., Xu, D., Yang, B., Xie, C., Fan, L., Gao, S., Xu, X., Zhang, G., Li, Y., Jiao, Y., Doebley, J. F., Ross-Ibarra, J., Lorant, A., Buffalo, V., Romay, M. C., Buckler, E. S., Ware, D., Lai, J., Sun, Q., and Xu, Y. (2017). Construction of the third-generation *Zea mays* haplotype map. *GigaScience*, 7(4).

Caetano-Anolles, D. (2023). Hard-filtering germline short variants.

Chamberlain, S. A. and Boettiger, C. (2017). R Python, and Ruby clients for GBIF species occurrence data. Technical Report e3304v1, PeerJ Inc. ISSN: 2167-9843.

Chen, J., Glémin, S., and Lascoux, M. (2017). Genetic diversity and the efficacy of purifying selection across plant and animal species. *Molecular Biology and Evolution*, 34(6):1417–1428.

Chen, S., Zhou, Y., Chen, Y., and Gu, J. (2018). fastp: an ultra-fast all-in-one FASTQ preprocessor. *Bioinformatics*, 34(17):i884–i890.

Corbett-Detig, R. B., Hartl, D. L., and Sackton, T. B. (2015). Natural selection constrains neutral diversity across a wide range of species. *PLOS Biology*, 13(4):e1002112. Publisher: Public Library of Science.

Danecek, P., Bonfield, J. K., Liddle, J., Marshall, J., Ohan, V., Pollard, M. O., Whitwham, A., Keane, T., McCarthy, S. A., Davies, R. M., and Li, H. (2021). Twelve years of SAMtools and BCFtools. *GigaScience*, 10(2):giab008.

Deng, J., Zuo, W., Wang, Z., Fan, Z., Ji, M., Wang, G., Ran, J., Zhao, C., Liu, J., Niklas, K. J., Hammond, S. T., and Brown, J. H. (2012). Insights into plant size-density relationships from models and agricultural crops. *Proceedings of the National Academy of Sciences*, 109(22):8600–8605. Publisher: Proceedings of the National Academy of Sciences.

Doležel, J., Bartoš, J., Voglmayr, H., and Greilhuber, J. (2003). Nuclear DNA content and genome size of trout and human. *Cytometry Part A*, 51A(2):127–128. _eprint: https://onlinelibrary.wiley.com/doi/pdf/10.1002/cyto.a.10013.

Dubinkina, V. B., Ischenko, D. S., Ulyantsev, V. I., Tyakht, A. V., and Alexeev, D. G. (2016). Assessment of k-mer spectrum applicability for metagenomic dissimilarity analysis. *BMC Bioinformatics*, 17(1):38.

Ewels, P., Magnusson, M., Lundin, S., and Käller, M. (2016). MultiQC: summarize analysis results for multiple tools and samples in a single report. *Bioinformatics*, 32(19):3047–3048.

Filatov, D. A. (2019). Extreme Lewontin's Paradox in Ubiquitous Marine Phytoplankton Species. *Molecular Biology and Evolution*, 36(1):4–14.

Fofanov, Y., Luo, Y., Katili, C., Wang, J., Belosludtsev, Y., Powdrill, T., Belapurkar, C., Fofanov, V., Li, T.-B., Chumakov, S., and Pettitt, B. M. (2004). How independent are the appearances of n-mers in different genomes? *Bioinformatics*, 20(15):2421–2428.

Glasenapp, M. R. and Pogson, G. H. (2024). Selection Shapes the Genomic Landscape of Introgressed Ancestry in a Pair of Sympatric Sea Urchin Species. *Genome Biology and Evolution*, 16(6):evae124.

Govaerts, R., Nic Lughadha, E., Black, N., Turner, R., and Paton, A. (2021). The World Checklist of Vascular Plants, a continuously updated resource for exploring global plant diversity. *Scientific Data*, 8(1):215. Number: 1 Publisher: Nature Publishing Group.

Guo, X., Wang, Y., Keightley, P. D., and Fan, L. (2007). Patterns of selective constraints in noncoding DNA of rice. *BMC Evolutionary Biology*, 7(1):208.

Hahn, M. W. (2018). *Molecular Population Genetics*. Oxford University Press. Google-Books-ID: 3BD-kswEACAAJ.

Halushka, M. K., Fan, J.-B., Bentley, K., Hsie, L., Shen, N., Weder, A., Cooper, R., Lipshutz, R., and Chakravarti, A. (1999). Patterns of single-nucleotide polymorphisms in candidate genes for blood-pressure homeostasis. *Nature Genetics*, 22(3):239–247. Publisher: Nature Publishing Group.

Institute, B. (2019). Picard toolkit. *Broad Institute, GitHub repository.*

Johnsson, P., Lipovich, L., Grandér, D., and Morris, K. V. (2014). Evolutionary conservation of long noncoding RNAs; sequence, structure, function. *Biochimica et biophysica acta*, 1840(3):1063–1071.

Kattge, J., Díaz, S., Lavorel, S., Prentice, I. C., Leadley, P., Bönisch, G., Garnier, E., Westoby, M., Reich, P. B., Wright, I. J., Cornelissen, J. H. C., Violle, C., Harrison, S. P., Van Bodegom, P. M., Reichstein, M., Enquist, B. J., Soudzilovskaia, N. A., Ackerly, D. D., Anand, M., Atkin, O., Bahn, M., Baker, T. R., Baldocchi, D., Bekker, R., Blanco, C. C., Blonder, B., Bond, W. J., Bradstock, R., Bunker, D. E.,

Casanoves, F., Cavender-Bares, J., Chambers, J. Q., Chapin Iii, F. S., Chave, J., Coomes, D., Cornwell, W. K., Craine, J. M., Dobrin, B. H., Duarte, L., Durka, W., Elser, J., Esser, G., Estiarte, M., Fagan, W. F., Fang, J., Fernández-Méndez, F., Fidelis, A., Finegan, B., Flores, O., Ford, H., Frank, D., Freschet, G. T., Fyllas, N. M., Gallagher, R. V., Green, W. A., Gutierrez, A. G., Hickler, T., Higgins, S. I., Hodgson, J. G., Jalili, A., Jansen, S., Joly, C. A., Kerkhoff, A. J., Kirkup, D., Kitajima, K., Kleyer, M., Klotz, S., Knops, J. M. H., Kramer, K., Kühn, I., Kurokawa, H., Laughlin, D., Lee, T. D., Leishman, M., Lens, F., Lenz, T., Lewis, S. L., Lloyd, J., Llusià, J., Louault, F., Ma, S., Mahecha, M. D., Manning, P., Massad, T., Medlyn, B. E., Messier, J., Moles, A. T., Müller, S. C., Nadrowski, K., Naeem, S., Niinemets, , Nöllert, S., Nüske, A., Ogaya, R., Oleksyn, J., Onipchenko, V. G., Onoda, Y., Ordoñez, J., Overbeck, G., Ozinga, W. A., Patiño, S., Paula, S., Pausas, J. G., Peñuelas, J., Phillips, O. L., Pillar, V., Poorter, H., Poorter, L., Poschlod, P., Prinzing, A., Proulx, R., Rammig, A., Reinsch, S., Reu, B., Sack, L., Salgado-Negret, B., Sardans, J., Shiodera, S., Shipley, B., Siefert, A., Sosinski, E., Soussana, J., Swaine, E., Swenson, N., Thompson, K., Thornton, P., Waldram, M., Weiher, E., White, M., White, S., Wright, S. J., Yguel, B., Zaehle, S., Zanne, A. E., and Wirth, C. (2011). TRY – a global database of plant traits. *Global Change Biology*, 17(9):2905–2935.

Kim, J.-H., Park, J.-S., Lee, C.-Y., Jeong, M.-G., Xu, J. L., Choi, Y., Jung, H.-W., and Choi, H.-K. (2020). Dissecting seed pigmentation-associated genomic loci and genes by employing dual approaches of reference-based and k-mer-based GWAS with 438 *Glycine* accessions. *PLOS ONE*, 15(12):e0243085. Publisher: Public Library of Science.

Kokot, M., Długosz, M., and Deorowicz, S. (2017). KMC 3: counting and manipulating k-mer statistics. *Bioinformatics*, 33(17):2759–2761. Publisher: Oxford Academic.

Korunes, K. L. and Samuk, K. (2021). pixy: Unbiased estimation of nucleotide diversity and divergence in the presence of missing data. *Molecular Ecology Resources*, 21(4):1359–1368. _eprint: https://onlinelibrary.wiley.com/doi/pdf/10.1111/1755-0998.13326.

Leffler, E. M., Bullaughey, K., Matute, D. R., Meyer, W. K., Ségurel, L., Venkat, A., Andolfatto, P., and Przeworski, M. (2012). Revisiting an old riddle: what determines genetic diversity levels within species? *PLOS Biology*, 10(9):e1001388. Publisher: Public Library of Science.

Li, H. (2013). Aligning sequence reads, clone sequences and assembly contigs with BWA-MEM. arXiv:1303.3997 [q-bio].

Li, H. and Durbin, R. (2009). Fast and accurate short read alignment with Burrows–Wheeler transform. *Bioinformatics*, 25(14):1754–1760.

Makałowski, W. and Boguski, M. S. (1998). Evolutionary parameters of the transcribed mammalian genome: An analysis of 2,820 orthologous rodent and human sequences. *Proceedings of the National Academy of Sciences*, 95(16):9407–9412. Publisher: Proceedings of the National Academy of Sciences.

Margulies, E. H., Blanchette, M., Program, N. C. S., Haussler, D., and Green, E. D. (2003). Identification and characterization of multi-species conserved sequences. *Genome Research*, 13(12):2507–2518. Company: Cold Spring Harbor Laboratory Press Distributor: Cold Spring Harbor Laboratory Press Institution: Cold Spring Harbor Laboratory Press Label: Cold Spring Harbor Laboratory Press Publisher: Cold Spring Harbor Lab.

Mattila, T. M., Tyrmi, J., Pyhäjärvi, T., and Savolainen, O. (2017). Genome-Wide Analysis of Colonization History and Concomitant Selection in Arabidopsis lyrata. *Molecular Biology and Evolution*, 34(10):2665–2677.

McKenna, A., Hanna, M., Banks, E., Sivachenko, A., Cibulskis, K., Kernytsky, A., Garimella, K., Altshuler, D., Gabriel, S., Daly, M., and DePristo, M. A. (2010). The Genome Analysis Toolkit: A MapReduce framework for analyzing next-generation DNA sequencing data. *Genome Research*, 20(9):1297–1303. Company: Cold Spring Harbor Laboratory Press Distributor: Cold Spring Harbor Laboratory Press Institution: Cold

Spring Harbor Laboratory Press Label: Cold Spring Harbor Laboratory Press Publisher: Cold Spring Harbor Lab.

Mirchandani, C. D., Shultz, A. J., Thomas, G. W. C., Smith, S. J., Baylis, M., Arnold, B., Corbett-Detig, R., Enbody, E., and Sackton, T. B. (2024). A fast, reproducible, high-throughput variant calling workflow for population genomics. *Molecular Biology and Evolution*, 41(1):msad270.

Moriyama, E. N. and Powell, J. R. (1996). Intraspecific nuclear DNA variation in Drosophila. *Molecular Biology and Evolution*, 13(1):261–277.

Nordborg, M., Hu, T. T., Ishino, Y., Jhaveri, J., Toomajian, C., Zheng, H., Bakker, E., Calabrese, P., Gladstone, J., Goyal, R., Jakobsson, M., Kim, S., Morozov, Y., Padhukasahasram, B., Plagnol, V., Rosenberg, N. A., Shah, C., Wall, J. D., Wang, J., Zhao, K., Kalbfleisch, T., Schulz, V., Kreitman, M., and Bergelson, J. (2005). The pattern of polymorphism in *Arabidopsis thaliana*. *PLOS Biology*, 3(7):e196. Publisher: Public Library of Science.

Ondov, B. D., Treangen, T. J., Melsted, P., Mallonee, A. B., Bergman, N. H., Koren, S., and Phillippy, A. M. (2016). Mash: fast genome and metagenome distance estimation using MinHash. *Genome Biology*, 17(1):132.

Opedal, H., Armbruster, W. S., Hansen, T. F., Holstad, A., Pélabon, C., Andersson, S., Campbell, D. R., Caruso, C. M., Delph, L. F., Eckert, C. G., Lankinen, , Walter, G. M., Ågren, J., and Bolstad, G. H. (2023). Evolvability and trait function predict phenotypic divergence of plant populations. *Proceedings of the National Academy of Sciences*, 120(1):e2203228120. Publisher: Proceedings of the National Academy of Sciences.

Parr, C. S., Wilson, N., Leary, P., Schulz, K., Lans, K., Walley, L., Hammock, J., Goddard, A., Rice, J., Studer, M., Holmes, J., and Corrigan, Jr., R. (2014). The Encyclopedia of Life v2: Providing Global Access to Knowledge About Life on Earth. *Biodiversity Data Journal*, 2:e1079.

Pateiro-Lopez, B. and Rodriguez-Casal, A. (2022). alphahull: Generalization of the convex hull of a sample of points in the plane.

Pebesma, E. (2018). Simple Features for R: Standardized support for spatial vector data. *The R Journal*, 10(1):439–446.

Pellicer, J. and Leitch, I. J. (2020). The plant DNA C-values database (release 7.1): an updated online repository of plant genome size data for comparative studies. *New Phytologist*, 226(2):301–305. _eprint: https://nph.onlinelibrary.wiley.com/doi/pdf/10.1111/nph.16261.

Phung, T. N., Huber, C. D., and Lohmueller, K. E. (2016). Determining the effect of natural selection on linked neutral divergence across species. *PLOS Genetics*, 12(8):e1006199. Publisher: Public Library of Science.

Poplin, R., Ruano-Rubio, V., DePristo, M. A., Fennell, T. J., Carneiro, M. O., Auwera, G. A. V. d., Kling, D. E., Gauthier, L. D., Levy-Moonshine, A., Roazen, D., Shakir, K., Thibault, J., Chandran, S., Whelan, C., Lek, M., Gabriel, S., Daly, M. J., Neale, B., MacArthur, D. G., and Banks, E. (2018). Scaling accurate genetic variant discovery to tens of thousands of samples. Pages: 201178 Section: New Results.

Ramirez-Ramirez, A. R., Bidot-Martínez, I., Mirzaei, K., Rasoamanalina Rivo, O. L., Menéndez-Grenot, M., Clapé-Borges, P., Espinosa-Lopez, G., and Bertin, P. (2024). Comparing the performances of SSR and SNP markers for population analysis in Theobroma cacao L., as alternative approach to validate a new ddRADseq protocol for cacao genotyping. *PLOS ONE*, 19(5):e0304753.

Ranallo-Benavidez, T. R., Jaron, K. S., and Schatz, M. C. (2020). GenomeScope 2.0 and Smudgeplot for reference-free profiling of polyploid genomes. *Nature Communications*, 11(1):1432. Publisher: Nature Publishing Group.

Ritter, E. J., Cousins, P., Quigley, M., Kile, A., Kenchanmane Raju, S. K., Chitwood, D. H., and Niederhuth, C. (2024). From buds to shoots: insights into grapevine development from the Witch's Broom bud sport. *BMC Plant Biology*, 24(1):283.

Roberts, M. D., Davis, O., Josephs, E. B., and Williamson, R. J. (2024). k-mer-based approaches to bridging pangenomics and population genetics. Version Number: 1.

Ruperao, P., Gandham, P., Odeny, D. A., Mayes, S., Selvanayagam, S., Thirunavukkarasu, N., Das, R. R., Srikanda, M., Gandhi, H., Habyarimana, E., Manyasa, E., Nebie, B., Deshpande, S. P., and Rathore, A. (2023). Exploring the sorghum race level diversity utilizing 272 sorghum accessions genomic resources. *Frontiers in Plant Science*, 14.

Shajii, A., Yorukoglu, D., William Yu, Y., and Berger, B. (2016). Fast genotyping of known SNPs through approximate k-mer matching. *Bioinformatics*, 32(17):i538–i544.

Siepel, A., Bejerano, G., Pedersen, J. S., Hinrichs, A. S., Hou, M., Rosenbloom, K., Clawson, H., Spieth, J., Hillier, L. W., Richards, S., Weinstock, G. M., Wilson, R. K., Gibbs, R. A., Kent, W. J., Miller, W., and Haussler, D. (2005). Evolutionarily conserved elements in vertebrate, insect, worm, and yeast genomes. *Genome Research*, 15(8):1034–1050. Company: Cold Spring Harbor Laboratory Press Distributor: Cold Spring Harbor Laboratory Press Institution: Cold Spring Harbor Laboratory Press Label: Cold Spring Harbor Laboratory Press Publisher: Cold Spring Harbor Lab.

South, A. (2011). rworldmap: A new R package for mapping global data. *The R Journal*, 3(1):35–43.

Tenaillon, M. I., Sawkins, M. C., Long, A. D., Gaut, R. L., Doebley, J. F., and Gaut, B. S. (2001). Patterns of DNA sequence polymorphism along chromosome 1 of maize (*Zea mays* ssp. mays L.). *Proceedings of the National Academy of Sciences*, 98(16):9161–9166. Publisher: Proceedings of the National Academy of Sciences.

VanWallendael, A. and Alvarez, M. (2022). Alignment-free methods for polyploid genomes: Quick and reliable genetic distance estimation. *Molecular Ecology Resources*, 22(2):612–622. _eprint: https://onlinelibrary.wiley.com/doi/pdf/10.1111/1755-0998.13499.

Voichek, Y. and Weigel, D. (2020). Identifying genetic variants underlying phenotypic variation in plants without complete genomes. *Nature Genetics*, 52(5):534–540. Number: 5 Publisher: Nature Publishing Group.

Wang, J., Street, N. R., Scofield, D. G., and Ingvarsson, P. K. (2016). Natural selection and recombination rate variation shape nucleotide polymorphism across the genomes of three related *Populus* species. *Genetics*, 202(3):1185–1200.

Williamson, R. J., Josephs, E. B., Platts, A. E., Hazzouri, K. M., Haudry, A., Blanchette, M., and Wright, S. I. (2014). Evidence for Widespread Positive and Negative Selection in Coding and Conserved Noncoding Regions of Capsella grandiflora. *PLOS Genetics*, 10(9):e1004622. Publisher: Public Library of Science.

Woolfe, A., Goodson, M., Goode, D. K., Snell, P., McEwen, G. K., Vavouri, T., Smith, S. F., North, P., Callaway, H., Kelly, K., Walter, K., Abnizova, I., Gilks, W., Edwards, Y. J. K., Cooke, J. E., and Elgar, G. (2005). Highly conserved non-coding sequences are associated with vertebrate development. *PLoS Biology*, 3(1):e7.

Yu, H., Zhang, K., Cheng, G., Mei, C., Wang, H., and Zan, L. (2024). Genome-wide analysis reveals genomic diversity and signatures of selection in Qinchuan beef cattle. *BMC Genomics*, 25(1):558.

Zwick, M. E., Cutler, D. J., and Chakravarti, A. (2000). Patterns of Genetic Variation in Mendelian and Complex Traits. *Annual Review of Genomics and Human Genetics*, 1(1):387–407.

2 Supplemental figures: Exploring relationships in data before outlier removal

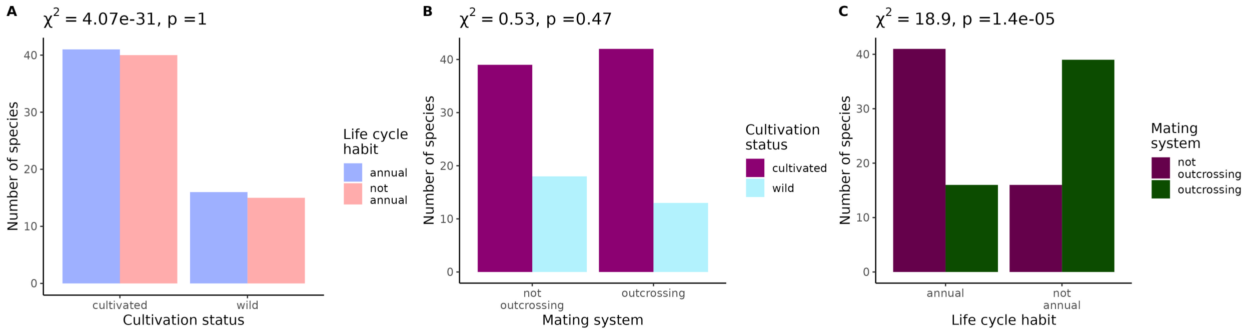

Figure S1: **Tests of independence between life-history traits included in this study.** Values across the top of each plot give the results of a χ^2 test of independence between each pair of life-history traits.

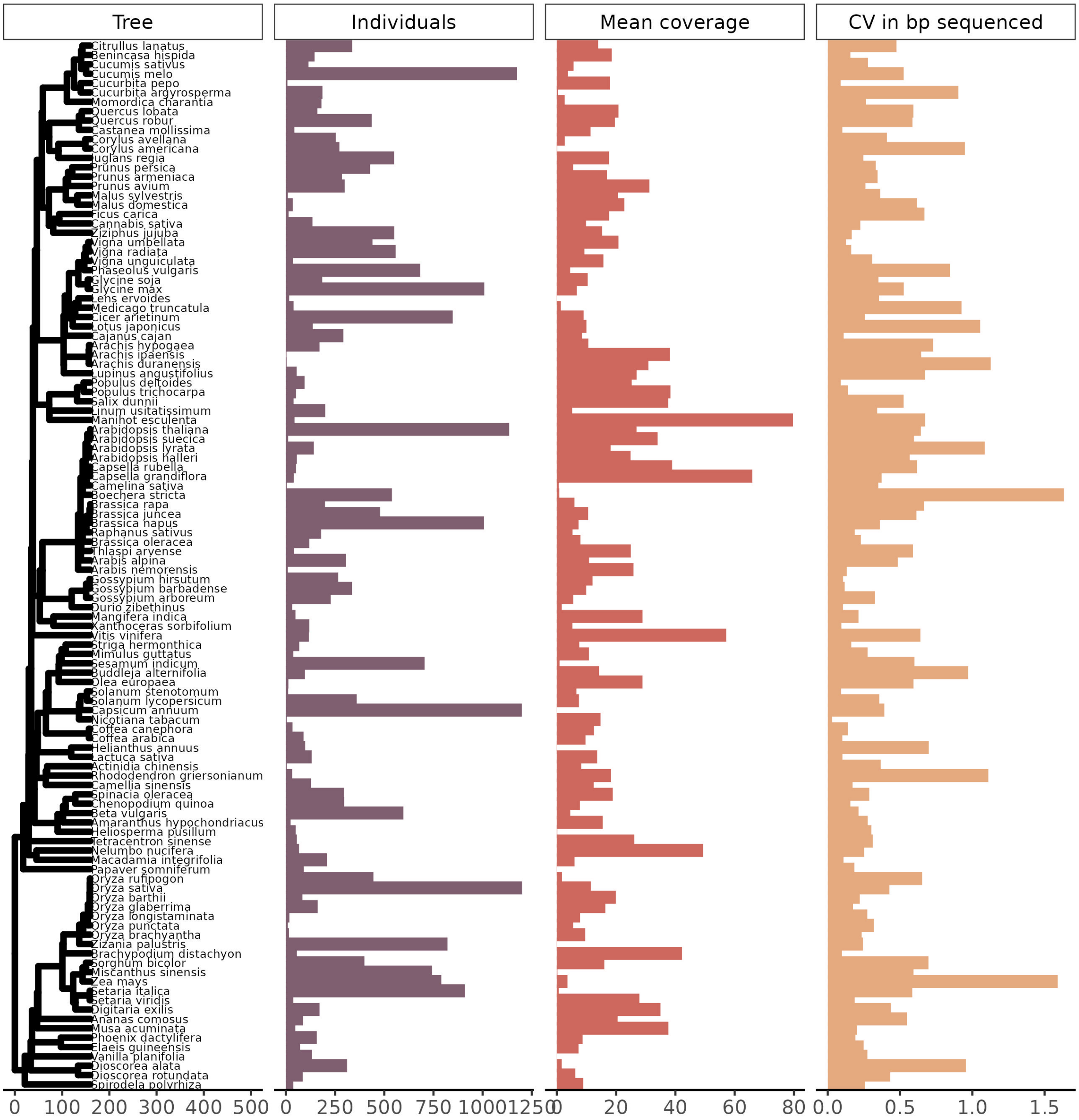

Figure S2: **Phylogenetic tree of species included in this study plotted alongside technical sequencing variables.** The phylogenetic tree scale is in millions of years. We limited the number of individuals sequenced per species to no more than 1200. CV stands for coefficient of variation and bp for base pairs.

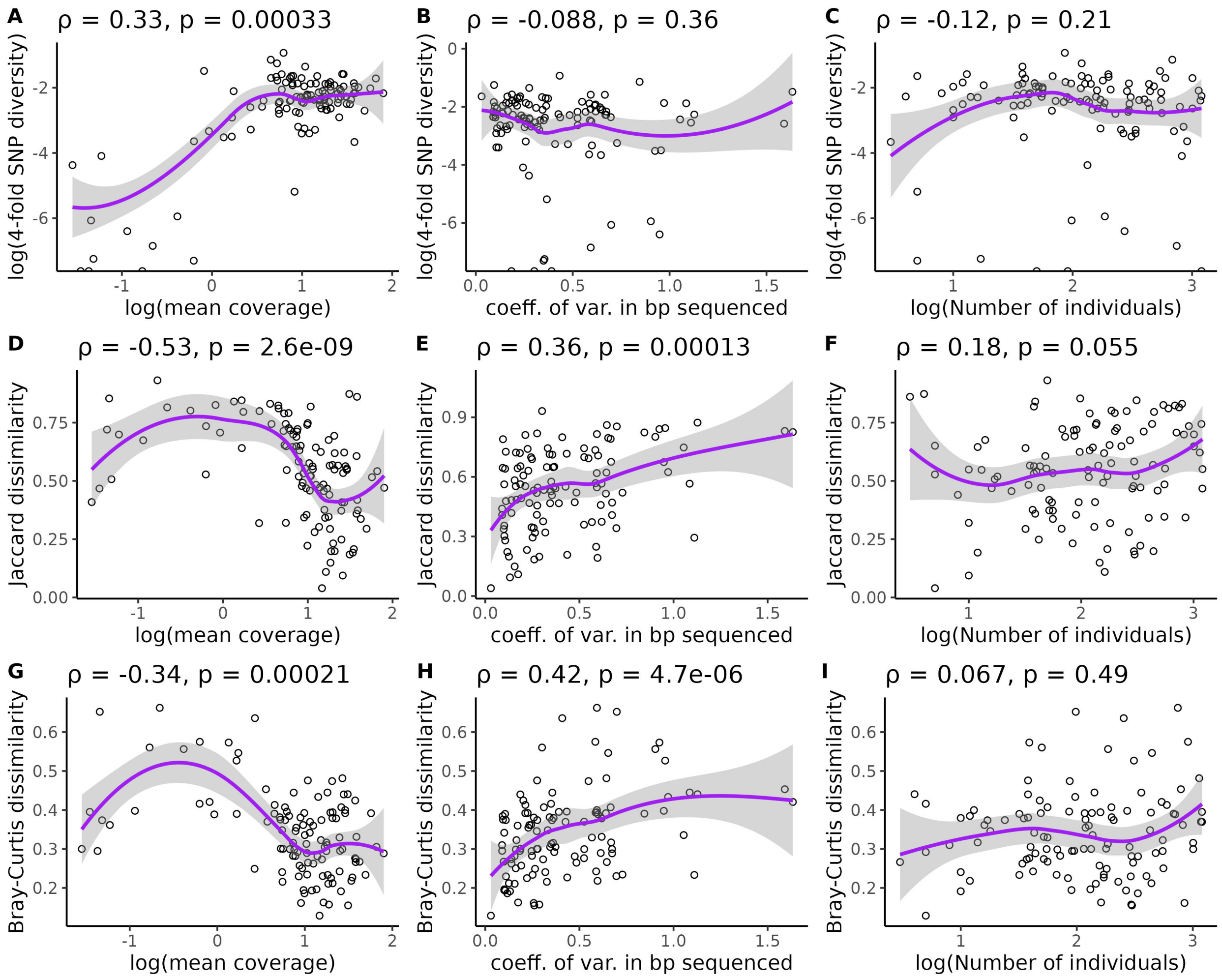

Figure S3: **Correlations between technical sequencing variables and diversity.** Each point is a species. Values across the top of each plot give the Spearman's correlation coefficient and p-value (testing whether the correlation differs from zero) for each pairwise relationship. Each pairwise plot includes one of three measures of diversity (Nucleotide diversity: A-C, Jaccard Dissimilarity: D-F, Bray-Curtis dissimilarity: G-I) and one of three technical sequencing variables (mean coverage: A, D, G; Coefficient of variation in bp sequenced: B, E, H; Number of individuals sequenced: C, F, I). Purple line is a loess smoothing line with 95% confidence intervals shaded in gray. All logarithms are base 10. Three species with nucleotide diversity values of 0 are omitted from plots involving nucleotide diversity.

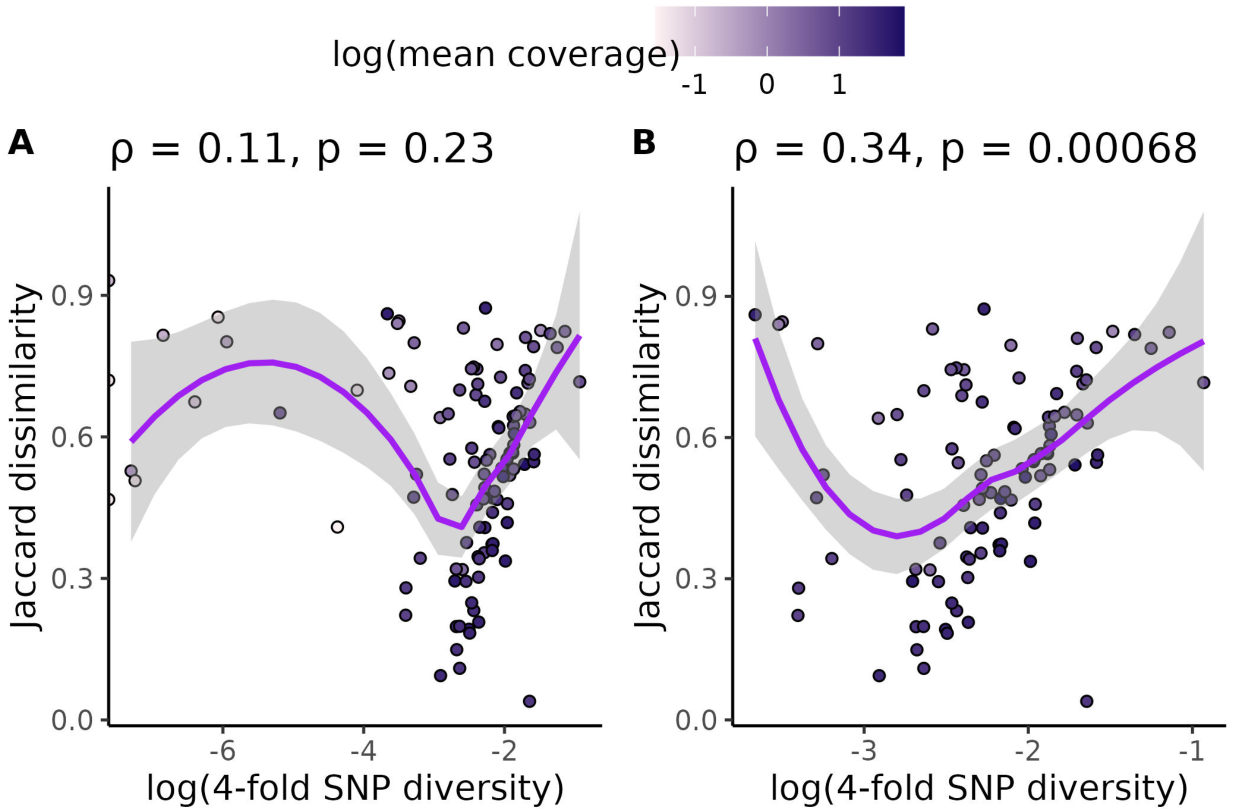

Figure S4: (A) shows the relationship between k-mer diversity (Jaccard dissimilarity) and nucleotide diversity without omitting species with ≤ 0.5x coverage or ≤ 1000 SNP calls. (B) shows the same relationship, except these species with low coverage or low numbers of SNP calls are omitted. Each data point is a species. All species' points are colored by the log of average genome-wide coverage per individual (base 10) for that species. Purple lines are loess smoothing curves with 95% confidence intervals shaded in gray. Values across the top of each plot are Spearman correlation coefficients (ρ) and p-values that test whether each correlation coefficient differs from zero. Three species with nucleotide diversity values of 0 are omitted from these plots.

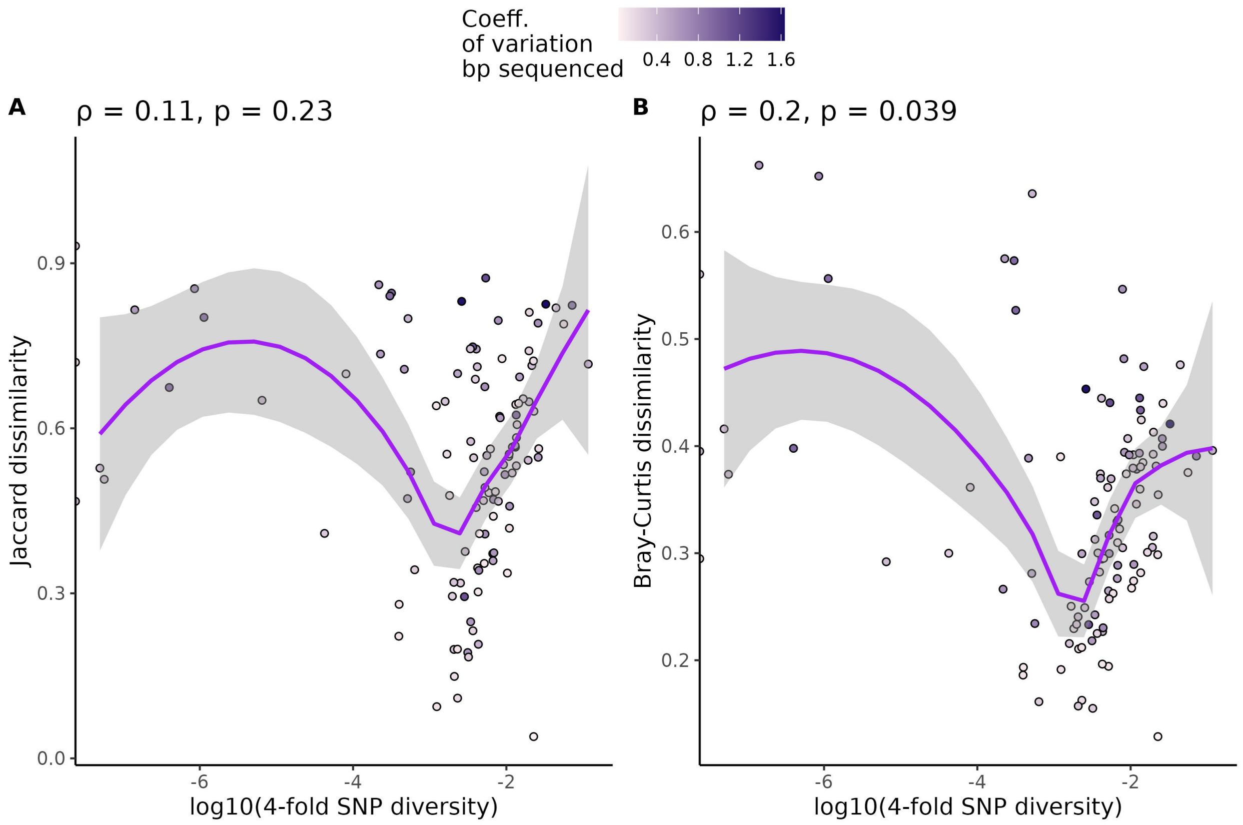

Figure S5: **Relationship between k-mer diversity, nucleotide diversity and variation in sequencing coverage.** Each point is a species. Values across the top of each plot give the Spearman's correlation coefficient and p-value (testing whether the correlation differs from zero) for each pairwise relationship. Purple line is a loess smoothing line with 95% confidence intervals shaded in gray. Points are colored by the coefficient of variation (mean/standard deviation) in bp sequenced for each species. Three species with nucleotide diversity values of 0 are omitted from the plots.

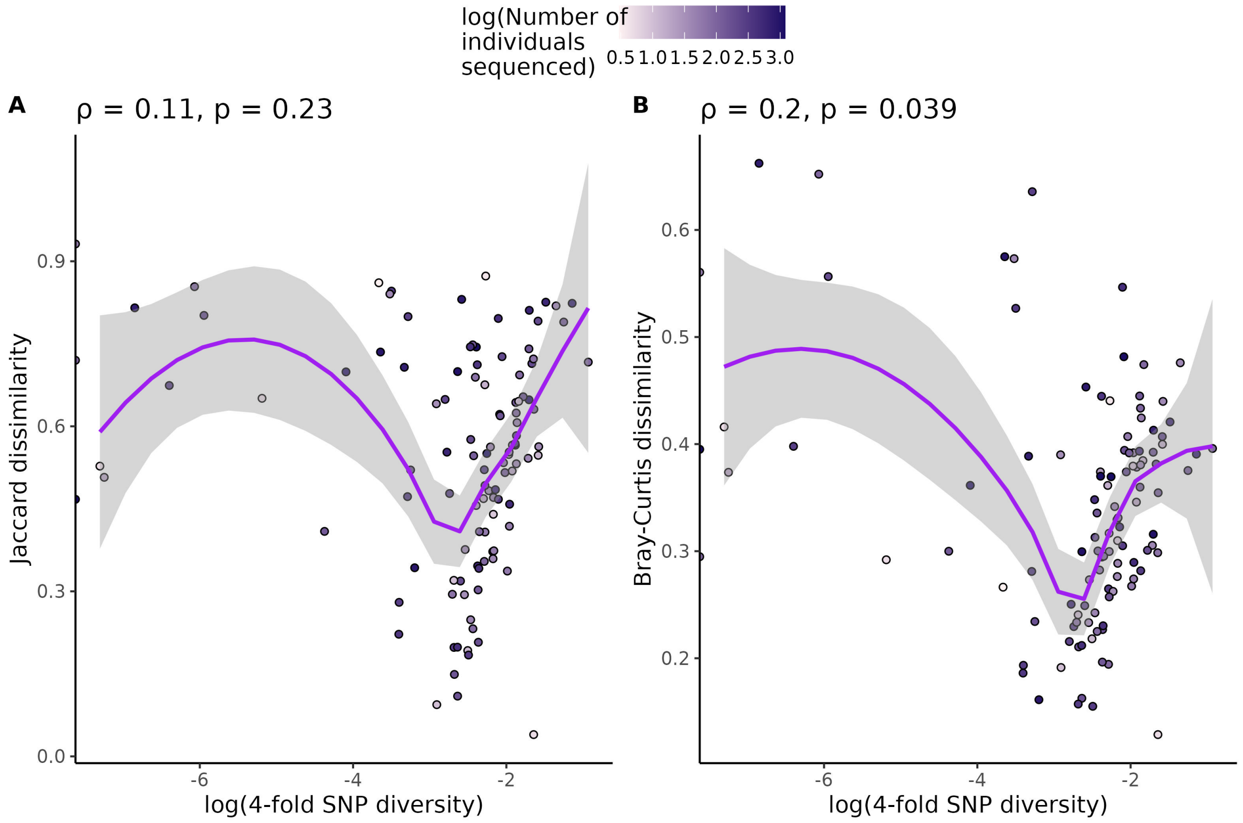

Figure S6: **Relationship between k-mer diversity, nucleotide diversity, and number of individuals sequenced.** Each point is a species. Values across the top of each plot give the Spearman's correlation coefficient and p-value (testing whether correlation differs from zero) for each pairwise relationship. Purple line is a loess smoothing line with 95% confidence intervals shaded in gray. Points are colored by the logarithm of the number of individuals sequenced within each species (base 10). Three species with nucleotide diversity values of 0 are omitted from the plots.

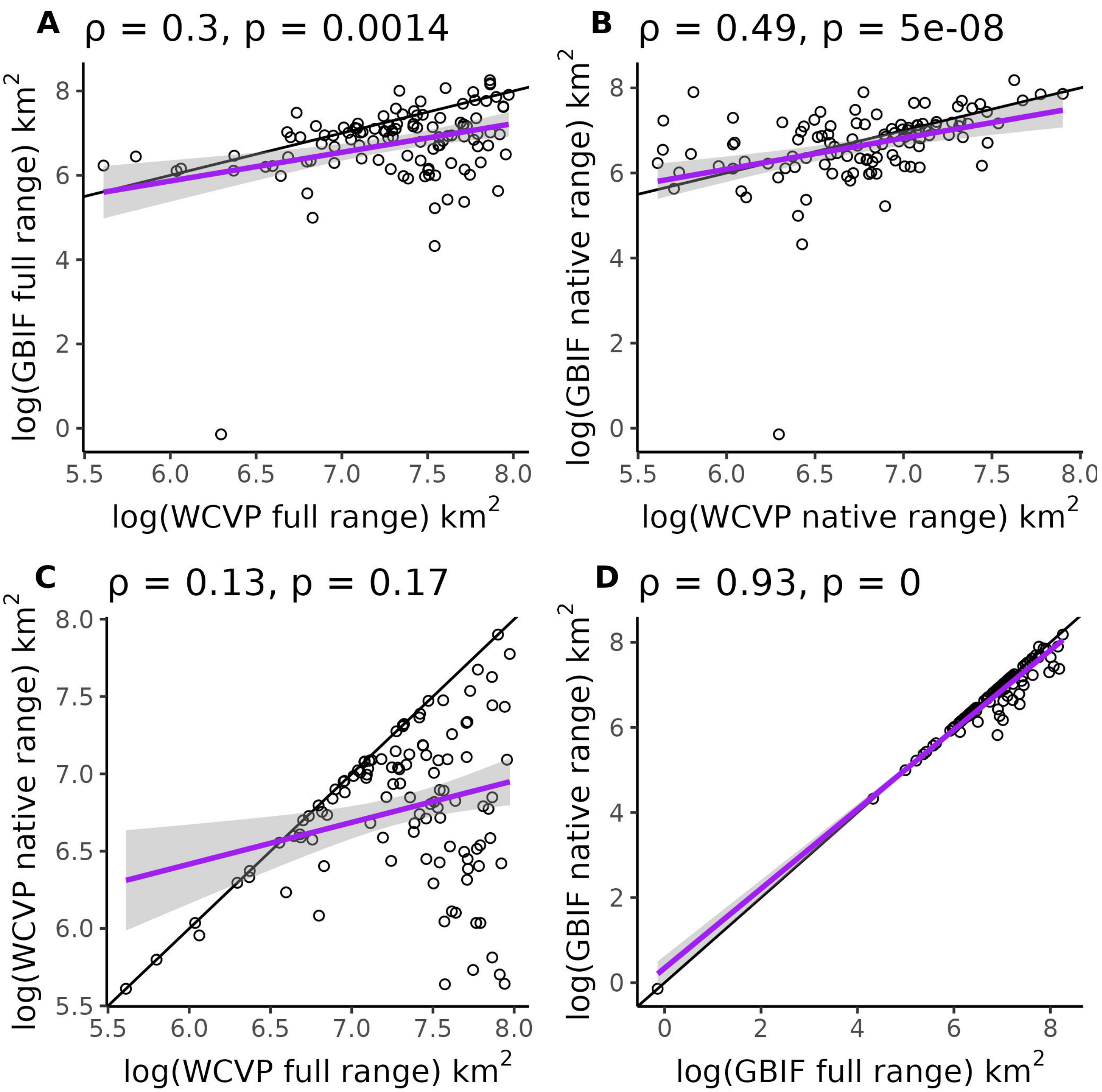

Figure S7: **Independent methods of range size estimation correlate with each other.** (A) Compares the size of total size of native and invaded ranges from GBIF occurrence data and WCVP range maps. (B) Compares native range estimates only (invaded ranges excluded) from GBIF occurrence data and WCVP range maps. (C) and (D) compare the full and native range estimates from WCVP range maps and GBIF occurrence data, respectively.Every data point is a species. The solid black lines are 1:1 reference lines where the different measures of range size are equal. The purple lines are linear regression lines with 95 % confidence intervals in grey shading. Values across the top of each plot give the Spearman's correlation coefficient (ρ) and p-value testing whether correlation differs from zero. One p-value is reported as zero because it was $< 2.2 \times 10^{-16}$, which is the limit of precision for doubles in R. All logarithms are base 10.

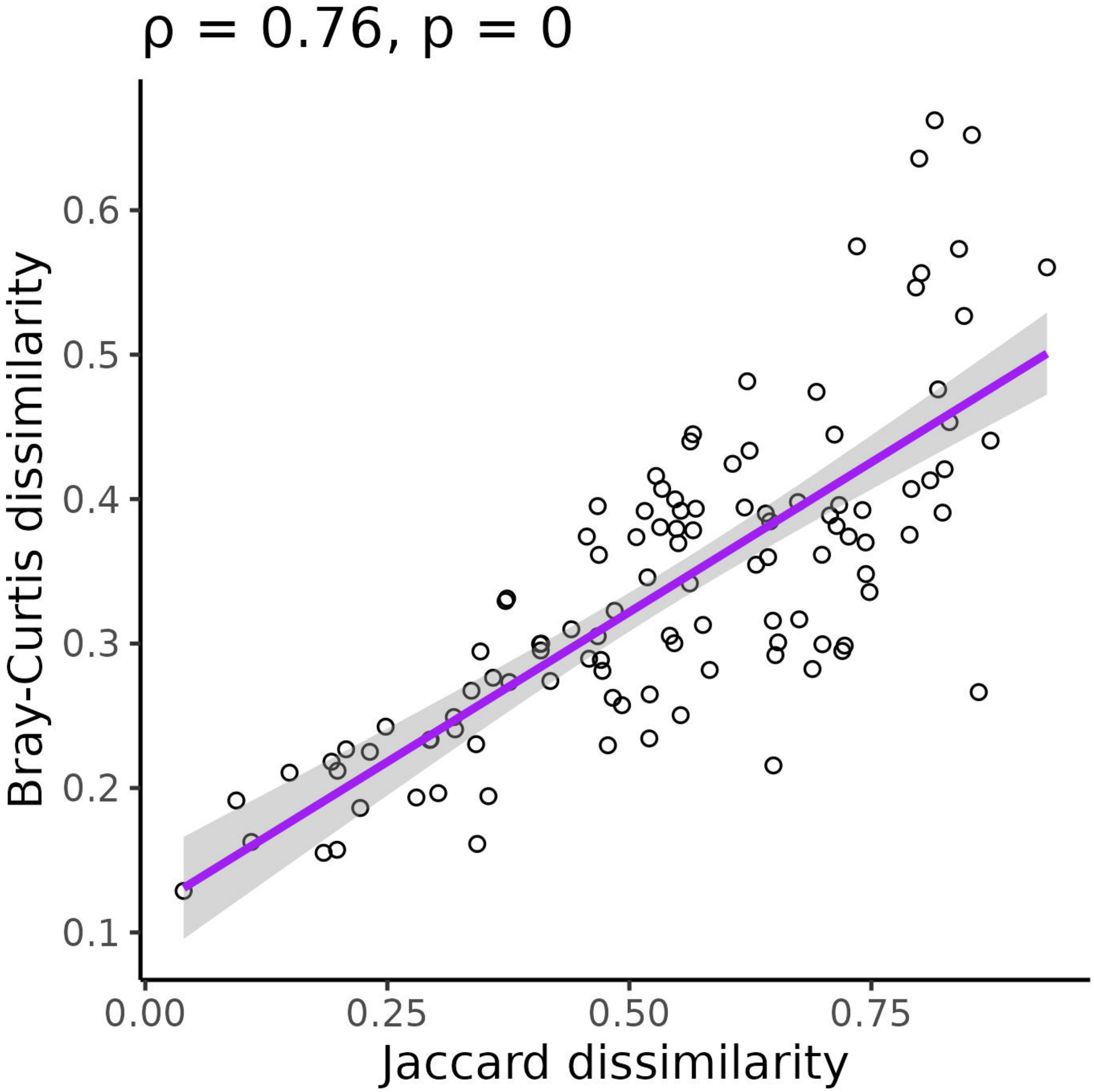

Figure S8: **Relationship between Bray-Curtis and Jaccard dissimilarity.** Each point is a species. Values across the top of each plot give the Spearman's correlation coefficient and p-value (testing whether correlation differs from zero). The p-value is reported as zero because it was $< 2.2 \times 10^{-16}$, which is the limit of precision for doubles in R. Line is a linear regression with 95% confidence intervals shaded in gray.

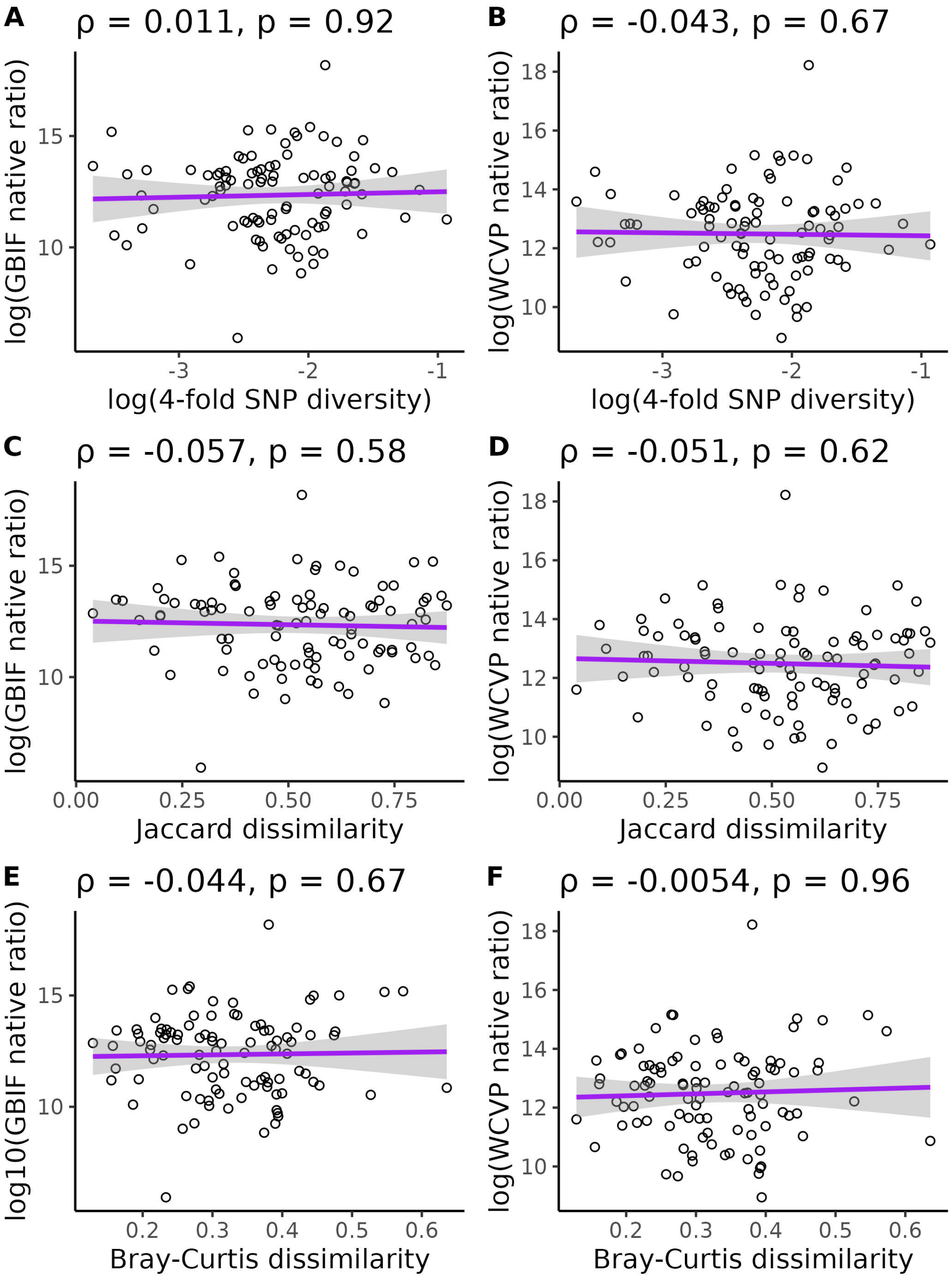

Figure S9: **Relationship between the range size-squared height ratio and diversity without correcting for evolutionary history, genome size, life cycle habit, mating system, or cultivation status.** Each point is a species. Values across the top of each plot give the Spearman's correlation coefficient and p-value (testing whether correlation differs from zero) for each pairwise relationship. Each pairwise relationship involves the native range size-squared height ratio, where native range size was estimated from GBIF occurrences (A, C, E), or WCVP range maps (B, D, F), and one of three diversity measures (Nucleotide diversity: A-B, Jaccard Dissimiarity: C-D, Bray-Curtis dissimilarity: E-F). Purple lines are loess smoothing lines with 95% confidence intervals shaded in gray. Three species with nucleotide diversity values of 0 are omitted from plots involving nucleotide diversity. All logarithms are base 10.

3 Supplemental figures: Population size proxy vs diversity relationships after controlling for phylogeny and life-history variables, but not controlling for genome size

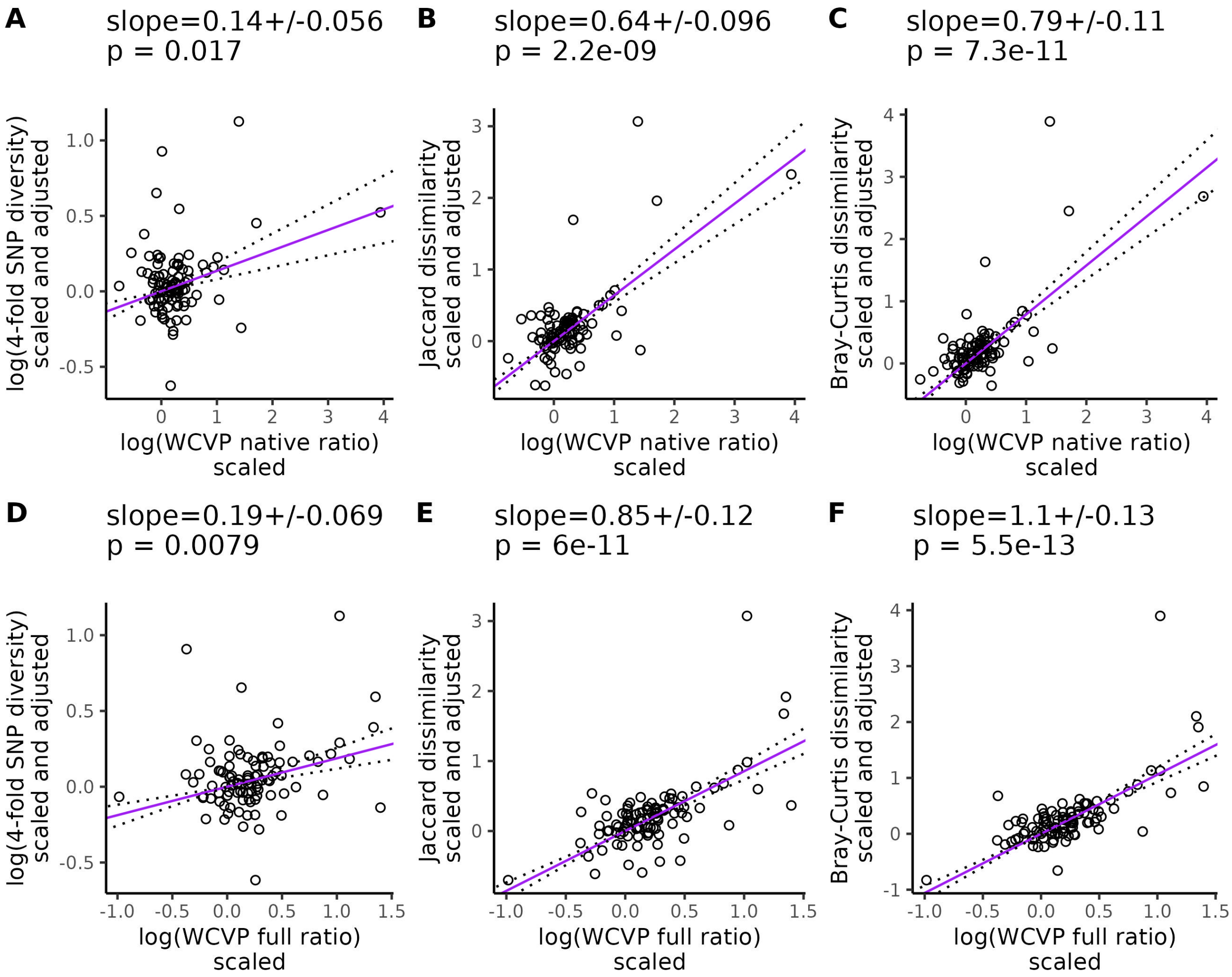

Figure S10: **Partial phylogenetic regression between WCVP population size proxy and diversity.** Each point is a species and only species with > 0.5x mean coverage and > 1000 variant sites were included in the regression. The regressions are organized according to whether invaded ranges were excluded (A-C) or included (D-F) in the range size-squared height ratio. Range size was estimated from WCVP range maps. Lines give the relationship between the pairs of plotted variables after controlling for mating system, life cycle habit, cultivation status, and evolutionary history. Before fitting the line, each response variable was scaled to a standard normal distribution (mean = 0, variance = 1), then multiplied by the inverse of the Cholesky decomposition of the phylogenetic variance-covariance matrix to correct for phylogenetic relationships. The values at the top of each plot give the slope of the partial regression ± one standard error and p-values testing whether the slopes differ from zero. Dotted lines show the partial regression slope ± one standard error. All logarithms are base 10.

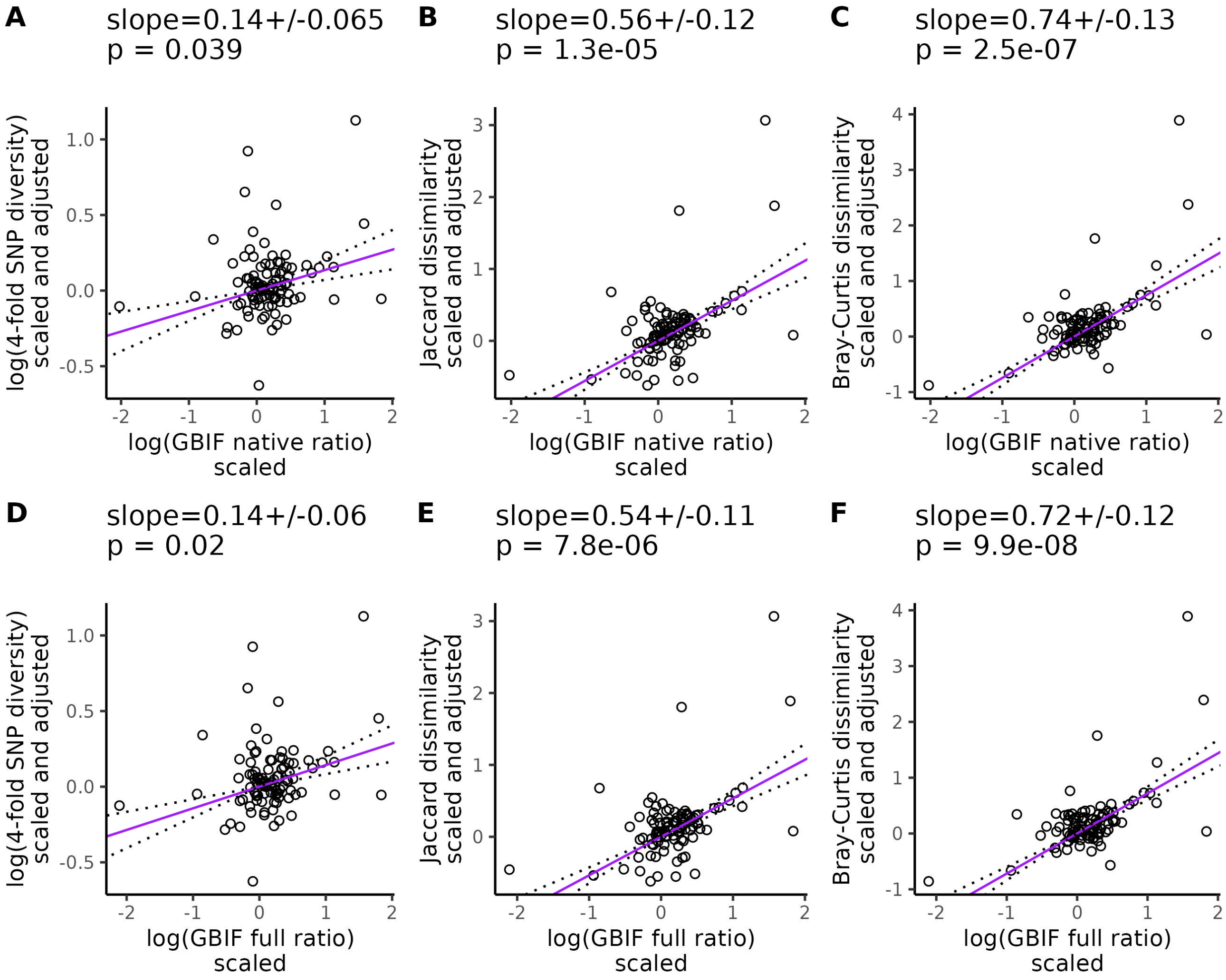

Figure S11: **Partial phylogenetic regression between GBIF population size proxy and diversity.** Each point is a species and only species with > 0.5x mean coverage and > 1000 variant sites were included in the regression. The regressions are organized according to whether invaded ranges were excluded (A-C) or included (D-F) in the range size-squared height ratio. Range size was estimated from GBIF occurrence data. Lines give the relationship between the pairs of plotted variables after controlling for mating system, life cycle habit, cultivation status, and evolutionary history. Before fitting the line, each response variable was scaled to a standard normal distribution (mean = 0, variance = 1), then multiplied by the inverse of the Cholesky decomposition of the phylogenetic variance-covariance matrix to correct for phylogenetic relationships. The values at the top of each plot give the slope of the partial regression ± one standard error and p-values testing whether the slopes differ from zero. Dotted lines show the partial regression slope ± one standard error. All logarithms are base 10.

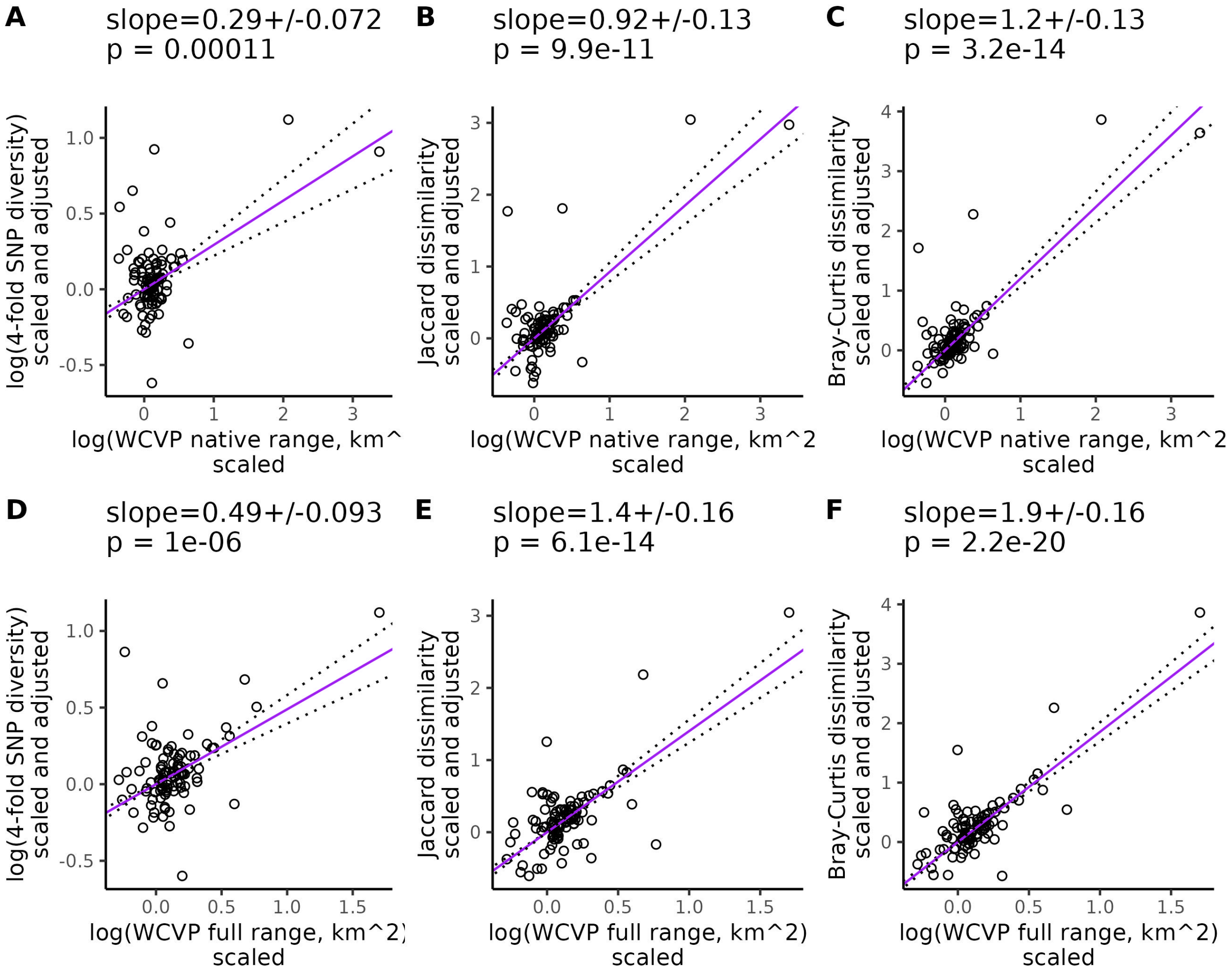

Figure S12: **Partial phylogenetic regression between WCVP range size and diversity.** Each point is a species and only species with > 0.5x mean coverage and > 1000 variant sites were included in the regression. The regressions are organized according to whether invaded ranges were excluded (A-C) or included (D-F) in the range size estimates. Range size was estimated from WCVP range maps. Lines give the relationship between the pairs of plotted variables after controlling for mating system, life cycle habit, cultivation status, and evolutionary history. Before fitting the line, each response variable was scaled to a standard normal distribution (mean = 0, variance = 1), then multiplied by the inverse of the Cholesky decomposition of the phylogenetic variance-covariance matrix to correct for phylogenetic relationships. The values at the top of each plot give the slope of the partial regression ± one standard error and p-values testing whether the slopes differ from zero. Dotted lines show the partial regression slope ± one standard error. All logarithms are base 10.

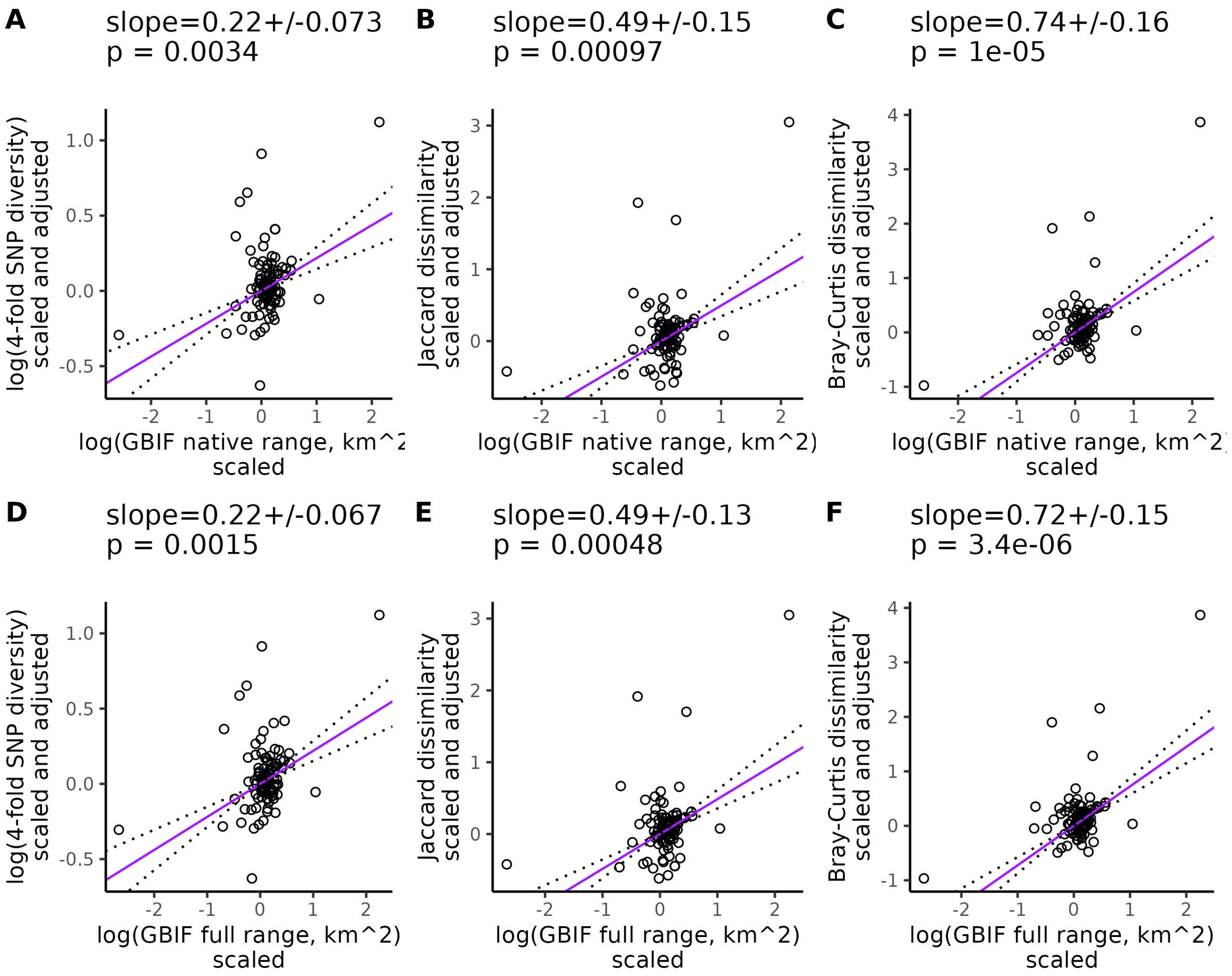

Figure S13: **Partial phylogenetic regression between GBIF range size and diversity.** Each point is a species and only species with > 0.5x mean coverage and > 1000 variant sites were included in the regression. The regressions are organized according to whether invaded ranges were excluded (A-C) or included (D-F) in the range size estimates. Range size was estimated from GBIF occurrence data. Lines give the relationship between the pairs of plotted variables after controlling for mating system, life cycle habit, cultivation status, and evolutionary history. Before fitting the line, each response variable was scaled to a standard normal distribution (mean = 0, variance = 1), then multiplied by the inverse of the Cholesky decomposition of the phylogenetic variance-covariance matrix to correct for phylogenetic relationships. The values at the top of each plot give the slope of the partial regression ± one standard error and p-values testing whether the slopes differ from zero. Dotted lines show the partial regression slope ± one standard error. All logarithms are base 10.

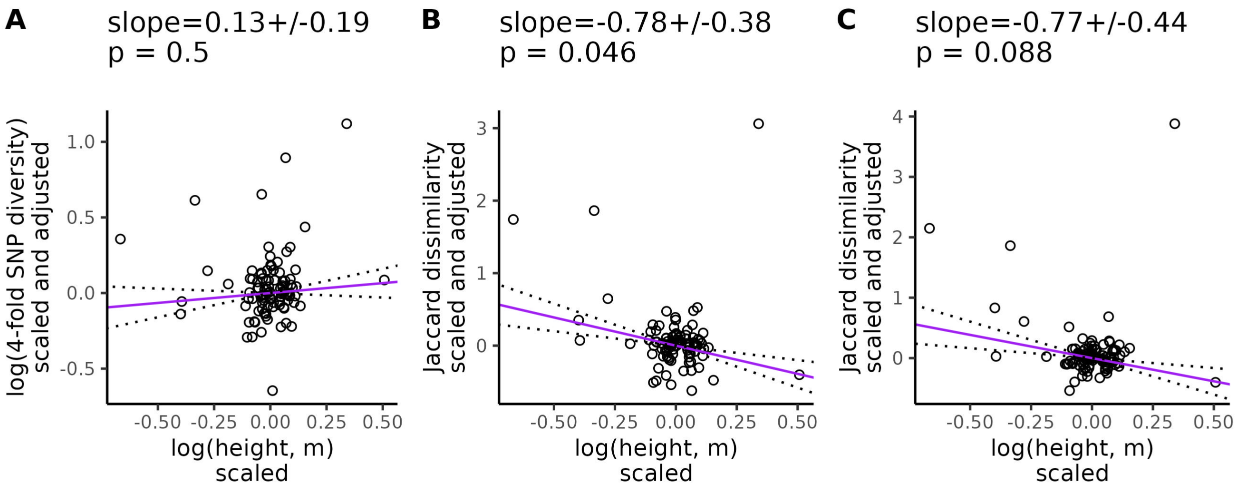

Figure S14: **Partial phylogenetic regression between height and diversity.** Each point is a species and only species with > 0.5x mean coverage and > 1000 variant sites were included in the regression. Lines give the relationship between the pairs of plotted variables after controlling for mating system, life cycle habit, cultivation status, and evolutionary history. The statistics across the top of each plot give the value of the slope of the lines ($\pm$ the standard error), the p-value testing whether the slope differs from zero. Before fitting the line, each response variable was scaled to a standard normal distribution (mean = 0, variance = 1), then multiplied by the inverse of the Cholesky decomposition of the phylogenetic variance-covariance matrix to correct for phylogenetic relationships. All logarithms are base 10.

4 Supplemental figures: Population size proxy vs diversity relationships after controlling for phylogeny, life-history variables, and genome size

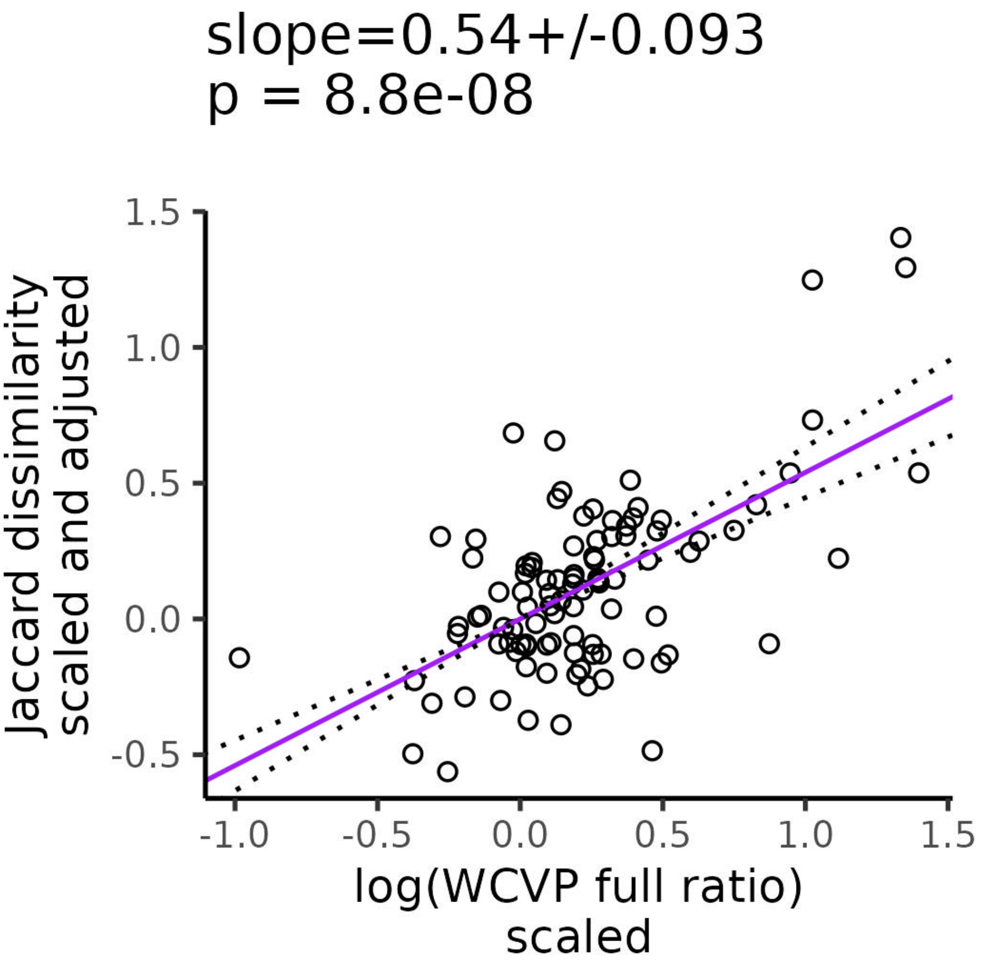

Figure S15: **Partial phylogenetic regression between WCVP full range size-squared height ratio and Jaccard dissimilarity, controlling for genome size.** Each point is a species and only species with > 0.5x mean coverage and > 1000 variant sites were included in the regression. WCVP full ratio gives the ratio of range size to squared plant height, where range size includes invaded ranges and is estimated from WCVP range maps. The partial regression controls for genome size, mating system, life cycle habit, cultivation status, and evolutionary history. Before fitting the line, each response variable was scaled to a standard normal distribution (mean = 0, variance = 1), then multiplied by the inverse of the Cholesky decomposition of the phylogenetic variance-covariance matrix to correct for phylogenetic relationships. The values at the top of the plot give the slope of the partial regression ± one standard error and p-values testing whether the slope differ from zero. Dotted lines show the partial regression slope ± one standard error. All logarithms are base 10.

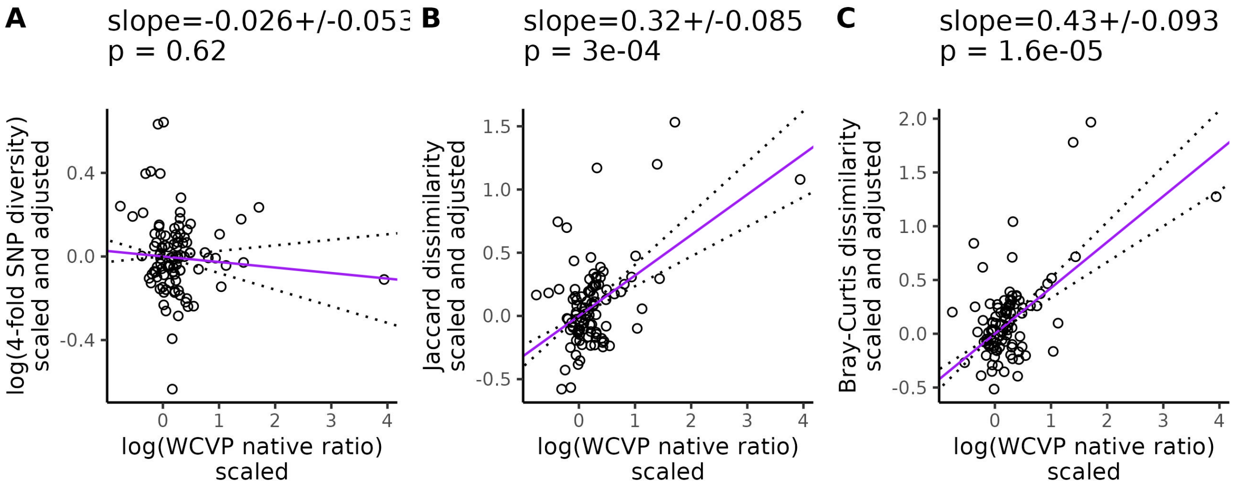

Figure S16: **Partial phylogenetic regression between WCVP native range size-squared height ratio and diversity, controlling for genome size.** Each point is a species and only species with > 0.5x mean coverage and > 1000 variant sites were included in the regression. WCVP native ratio gives the ratio of range size to squared plant height, where range size excludes invaded ranges and is estimated from WCVP range maps. The partial regression controls for genome size, mating system, life cycle habit, cultivation status, and evolutionary history. Before fitting the line, each response variable was scaled to a standard normal distribution (mean = 0, variance = 1), then multiplied by the inverse of the Cholesky decomposition of the phylogenetic variance-covariance matrix to correct for phylogenetic relationships. The values at the top of the plot give the slope of the partial regression $\pm$ one standard error and p-values testing whether the slope differ from zero. Dotted lines show the partial regression slope $\pm$ one standard error. All logarithms are base 10.

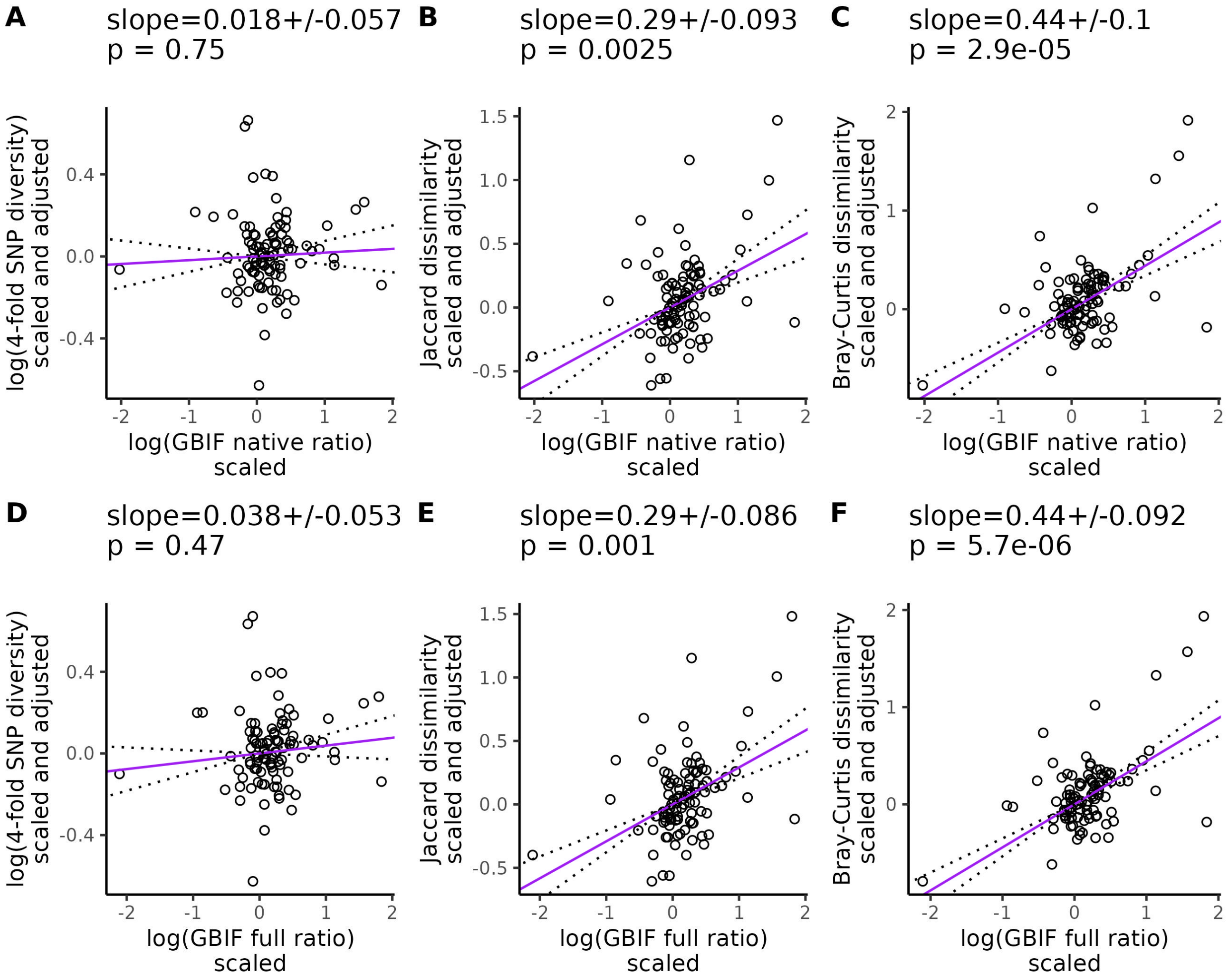

Figure S17: **Partial phylogenetic regression between GBIF range size-squared height ratio and diversity, controlling for genome size.** Each point is a species and only species with > 0.5x mean coverage and > 1000 variant sites were included in the regression. The regressions are organized according to whether invaded ranges were excluded (A-C) or included (D-F) in the range size-squared height ratio. Lines give the relationship between the pairs of plotted variables after controlling for mating system, life cycle habit, cultivation status, genome size, and evolutionary history. The statistics across the top of each plot give the value of the slope of the lines (± the standard error) and the p-value testing whether the slope differs from zero. Before fitting the line, each response variable was scaled to a standard normal distribution (mean = 0, variance = 1), then multiplied by the inverse of the Cholesky decomposition of the phylogenetic variance-covariance matrix to correct for phylogenetic relationships. Dotted lines show the partial regression slope ± one standard error. All logarithms are base 10.

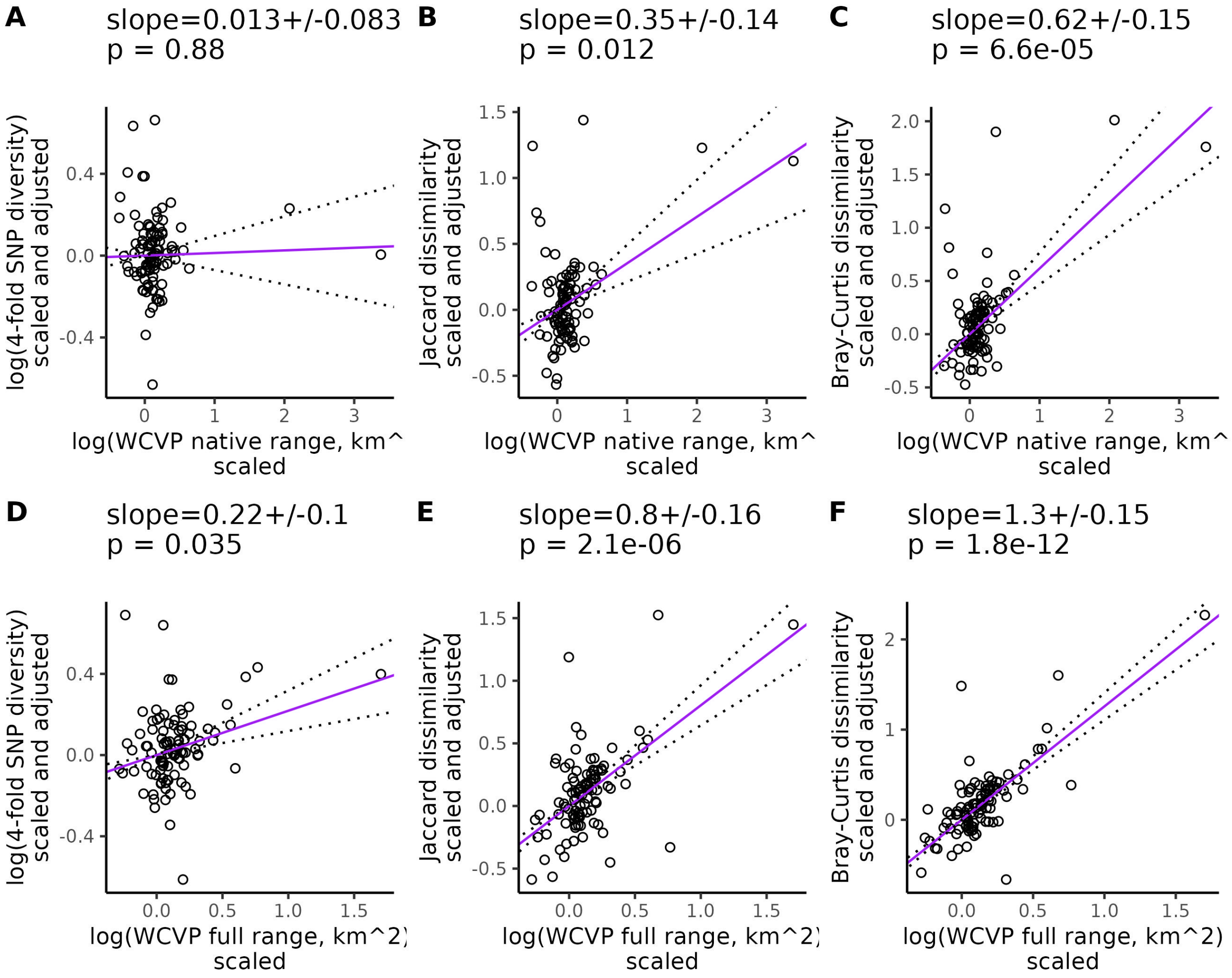

Figure S18: **Partial phylogenetic regression between WCVP range size and diversity, controlling for genome size.** Each point is a species and only species with > 0.5x mean coverage and > 1000 variant sites were included in the regression. The regressions are organized according to whether invaded ranges were excluded (A-C) or included (D-F) in the range size estimates. Lines give the relationship between the pairs of plotted variables after controlling for mating system, life cycle habit, cultivation status, genome size, and evolutionary history. The statistics across the top of each plot give the value of the slope of the lines (± the standard error) and the p-value testing whether the slope differs from zero. Before fitting the line, each response variable was scaled to a standard normal distribution (mean = 0, variance = 1), then multiplied by the inverse of the Cholesky decomposition of the phylogenetic variance-covariance matrix to correct for phylogenetic relationships. Dotted lines show the partial regression slope ± one standard error. All logarithms are base 10.

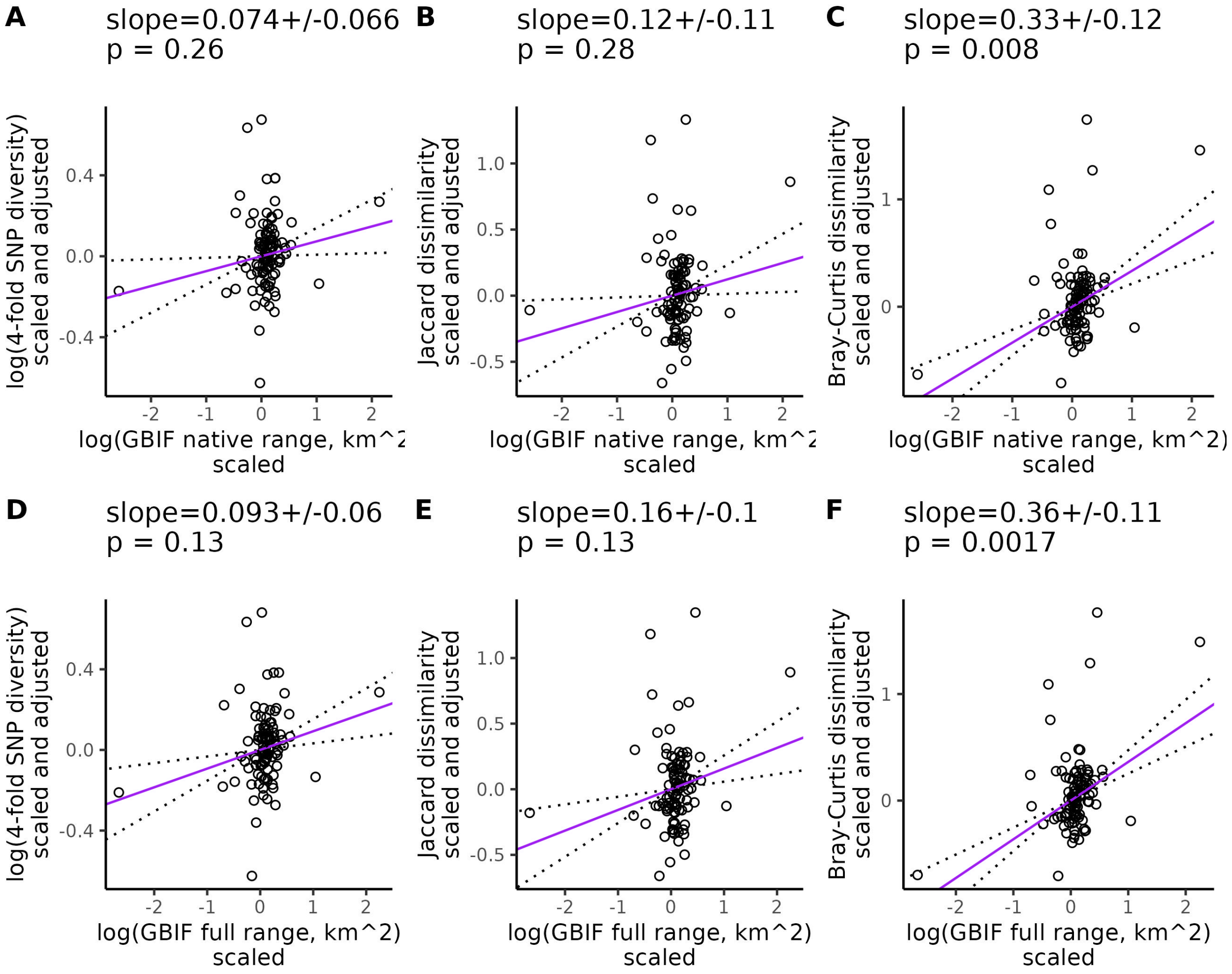

Figure S19: **Partial phylogenetic regression between GBIF range size and diversity, controlling for genome size.** Each point is a species and only species with $>$ 0.5x mean coverage and $>$ 1000 variant sites were included in the regression. The regressions are organized according to whether invaded ranges were excluded (A-C) or included (D-F) in the range size estimates. Lines give the relationship between the pairs of plotted variables after controlling for mating system, life cycle habit, cultivation status, genome size, and evolutionary history. The statistics across the top of each plot give the value of the slope of the lines ($\pm$ the standard error) and the p-value testing whether the slope differs from zero. Before fitting the line, each response variable was scaled to a standard normal distribution (mean = 0, variance = 1), then multiplied by the inverse of the Cholesky decomposition of the phylogenetic variance-covariance matrix to correct for phylogenetic relationships. Dotted lines show the partial regression slope $\pm$ one standard error. All logarithms are base 10.

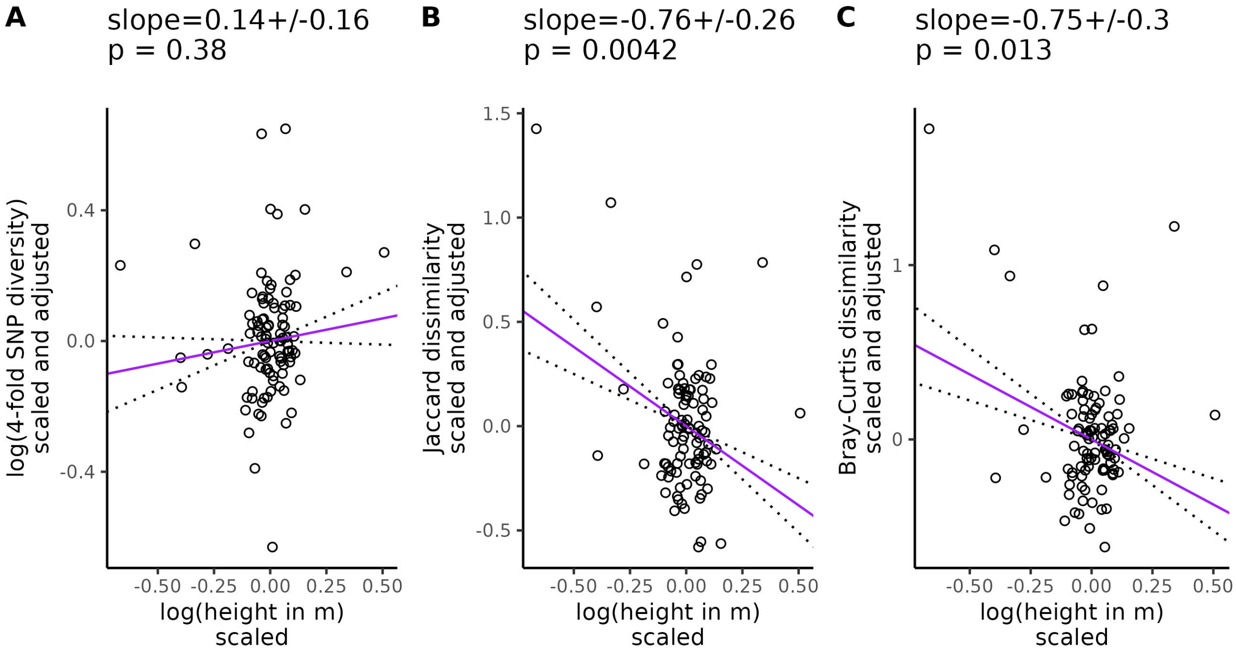

Figure S20: **Partial phylogenetic regression between height and diversity, controlling for genome size.** Each point is a species and only species with > 0.5x mean coverage and > 1000 variant sites were included in the regression. Lines give the relationship between the pairs of plotted variables after controlling for mating system, life cycle habit, cultivation status, genome size, and evolutionary history. The statistics across the top of each plot give the value of the slope of the lines ($\pm$ the standard error) and the p-value testing whether the slope differs from zero. Before fitting the line, each response variable was scaled to a standard normal distribution (mean = 0, variance = 1), then multiplied by the inverse of the Cholesky decomposition of the phylogenetic variance-covariance matrix to correct for phylogenetic relationships. Dotted lines show the partial regression slope $\pm$ one standard error. All logarithms are base 10.

5 Supplemental figures: Genome size vs diversity relationships after controlling for population size proxies, phylogeny, and life-history variables

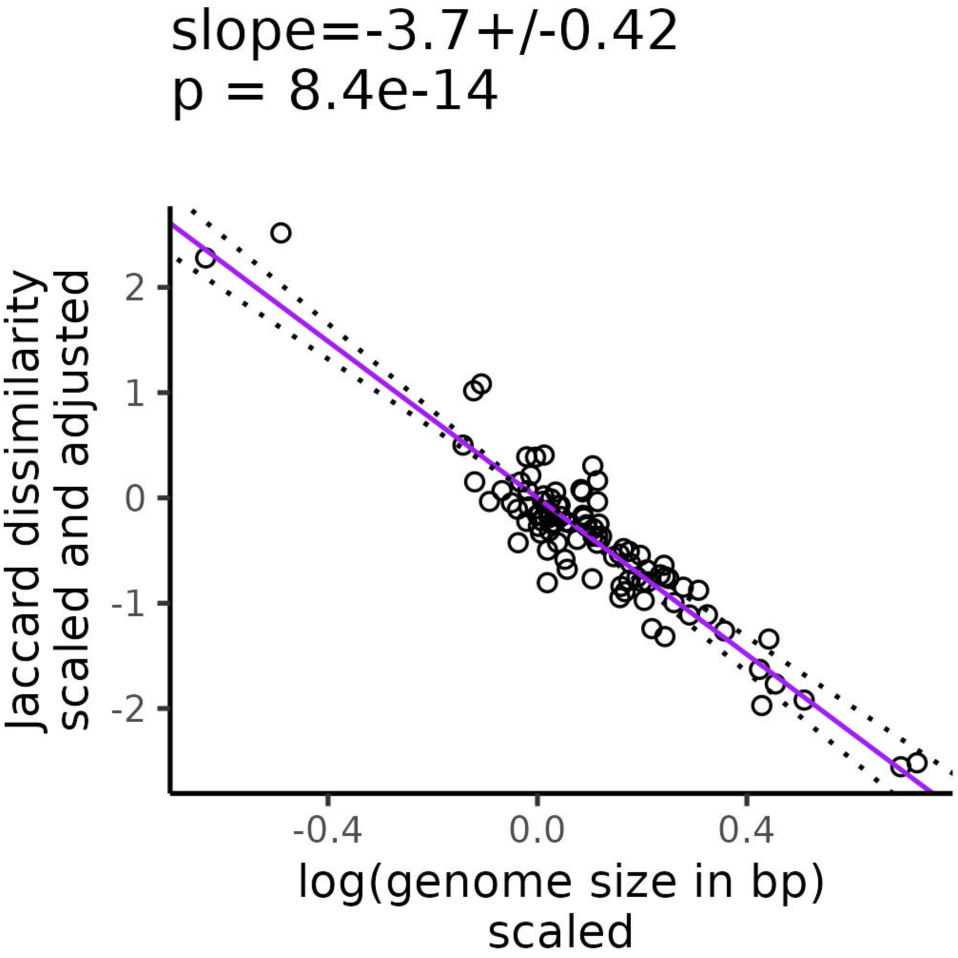

Figure S21: **Partial phylogenetic regression between Jaccard dissimilarity and genome size, controlling for WCVP full range size-height ratio.** Each point is a species and only species with > 0.5x mean coverage and > 1000 variant sites were included in the regression. WCVP full ratio gives the ratio of range size to squared plant height, where range size includes invaded ranges and is estimated from WCVP range maps. The partial regression controls for range size-squared height ratio, mating system, life cycle habit, cultivation status, and evolutionary history. Before fitting the line, each response variable was scaled to a standard normal distribution (mean = 0, variance = 1), then multiplied by the inverse of the Cholesky decomposition of the phylogenetic variance-covariance matrix to correct for phylogenetic relationships. The values at the top of the plot give the slope of the partial regression ± one standard error and p-values testing whether the slope differ from zero. Dotted lines show the partial regression slope ± one standard error. All logarithms are base 10.

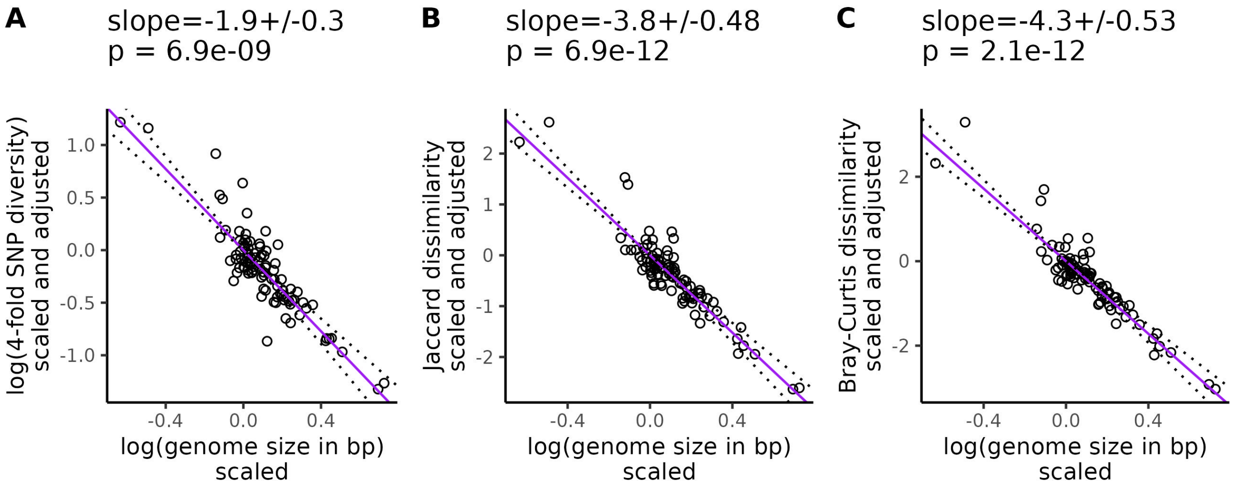

Figure S22: **Partial phylogenetic regression between diversity and genome size, controlling for WCVP native range size-height ratio.** Each point is a species and only species with $>$ 0.5x mean coverage and $>$ 1000 variant sites were included in the regression. WCVP native ratio gives the ratio of range size to squared plant height, where range size excludes invaded ranges and is estimated from WCVP range maps. The partial regression controls for genome size, mating system, life cycle habit, cultivation status, and evolutionary history. Before fitting the line, each response variable was scaled to a standard normal distribution (mean = 0, variance = 1), then multiplied by the inverse of the Cholesky decomposition of the phylogenetic variance-covariance matrix to correct for phylogenetic relationships. The values at the top of the plot give the slope of the partial regression $\pm$ one standard error and p-values testing whether the slope differ from zero. Dotted lines show the partial regression slope $\pm$ one standard error. All logarithms are base 10.

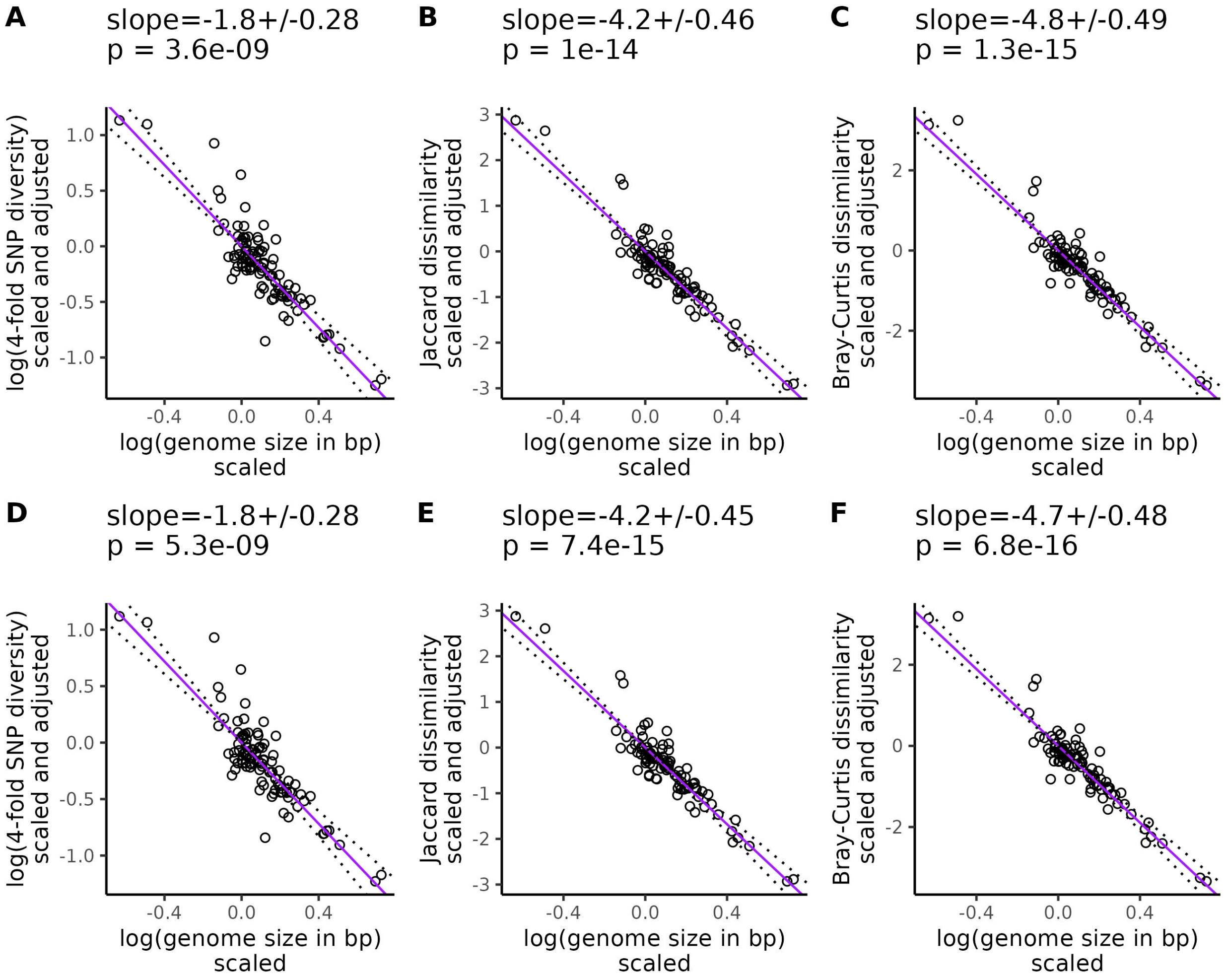

Figure S23: **Partial phylogenetic regression between diversity and genome size, controlling for GBIF range size-height ratio.** Each point is a species and only species with > 0.5x mean coverage and > 1000 variant sites were included in the regression. The regressions are organized according to whether invaded ranges were excluded (A-C) or included (D-F) in the range size-squared height ratio. Lines give the relationship between the pairs of plotted variables after controlling for mating system, life cycle habit, cultivation status, range size-squared height ratio, and evolutionary history. The statistics across the top of each plot give the value of the slope of the lines (± the standard error) and the p-value testing whether the slope differs from zero. Before fitting the line, each response variable was scaled to a standard normal distribution (mean = 0, variance = 1), then multiplied by the inverse of the Cholesky decomposition of the phylogenetic variance-covariance matrix to correct for phylogenetic relationships. Dotted lines show the partial regression slope ± one standard error. All logarithms are base 10.

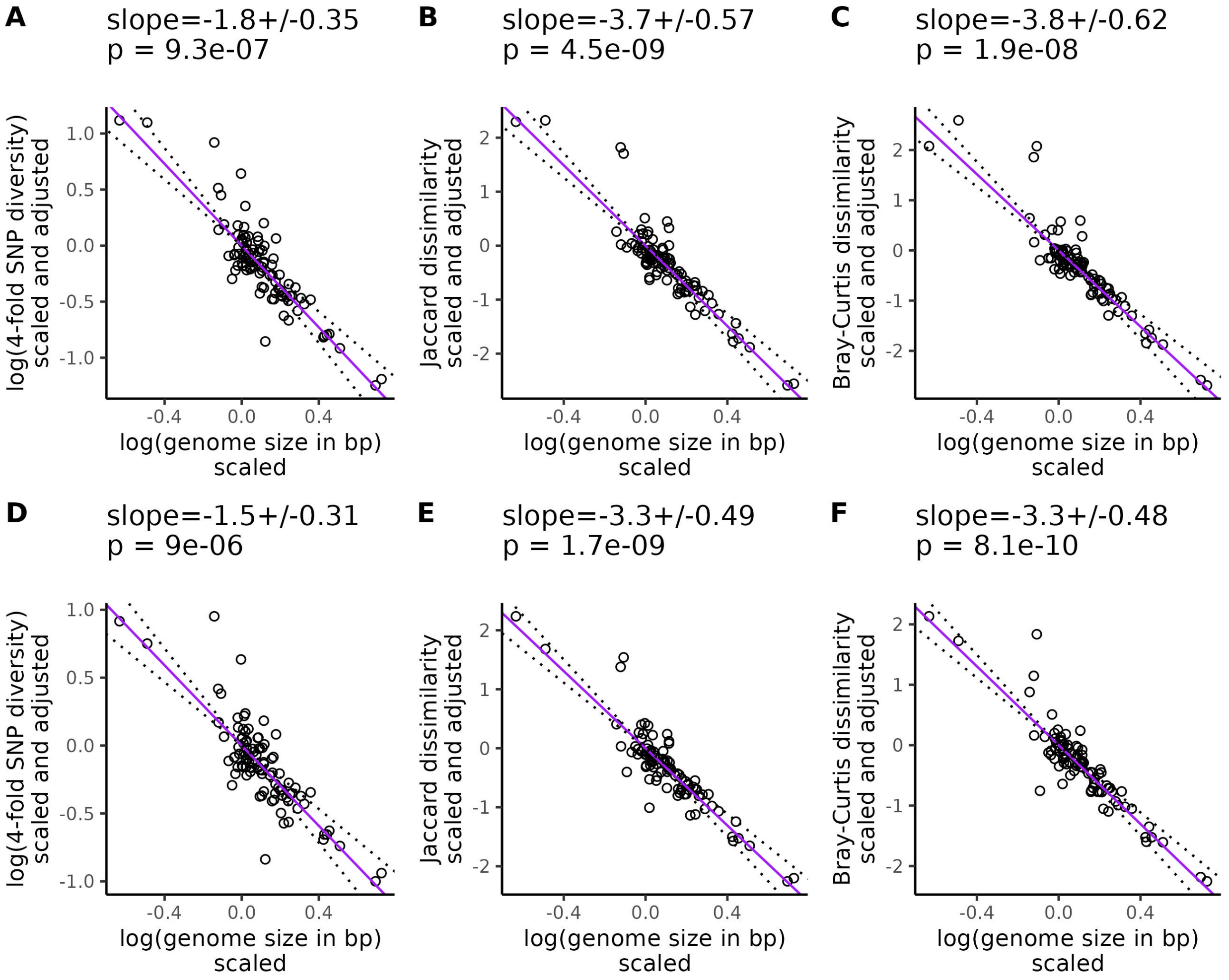

Figure S24: **Partial phylogenetic regression between diversity and genome size, controlling for WCVP range size.** Each point is a species and only species with > 0.5x mean coverage and > 1000 variant sites were included in the regression. The regressions are organized according to whether invaded ranges were excluded (A-C) or included (D-F) in the range size estimates. Lines give the relationship between the pairs of plotted variables after controlling for mating system, life cycle habit, cultivation status, range size, and evolutionary history. The statistics across the top of each plot give the value of the slope of the lines (± the standard error) and the p-value testing whether the slope differs from zero. Before fitting the line, each response variable was scaled to a standard normal distribution (mean = 0, variance = 1), then multiplied by the inverse of the Cholesky decomposition of the phylogenetic variance-covariance matrix to correct for phylogenetic relationships. Dotted lines show the partial regression slope ± one standard error. All logarithms are base 10.

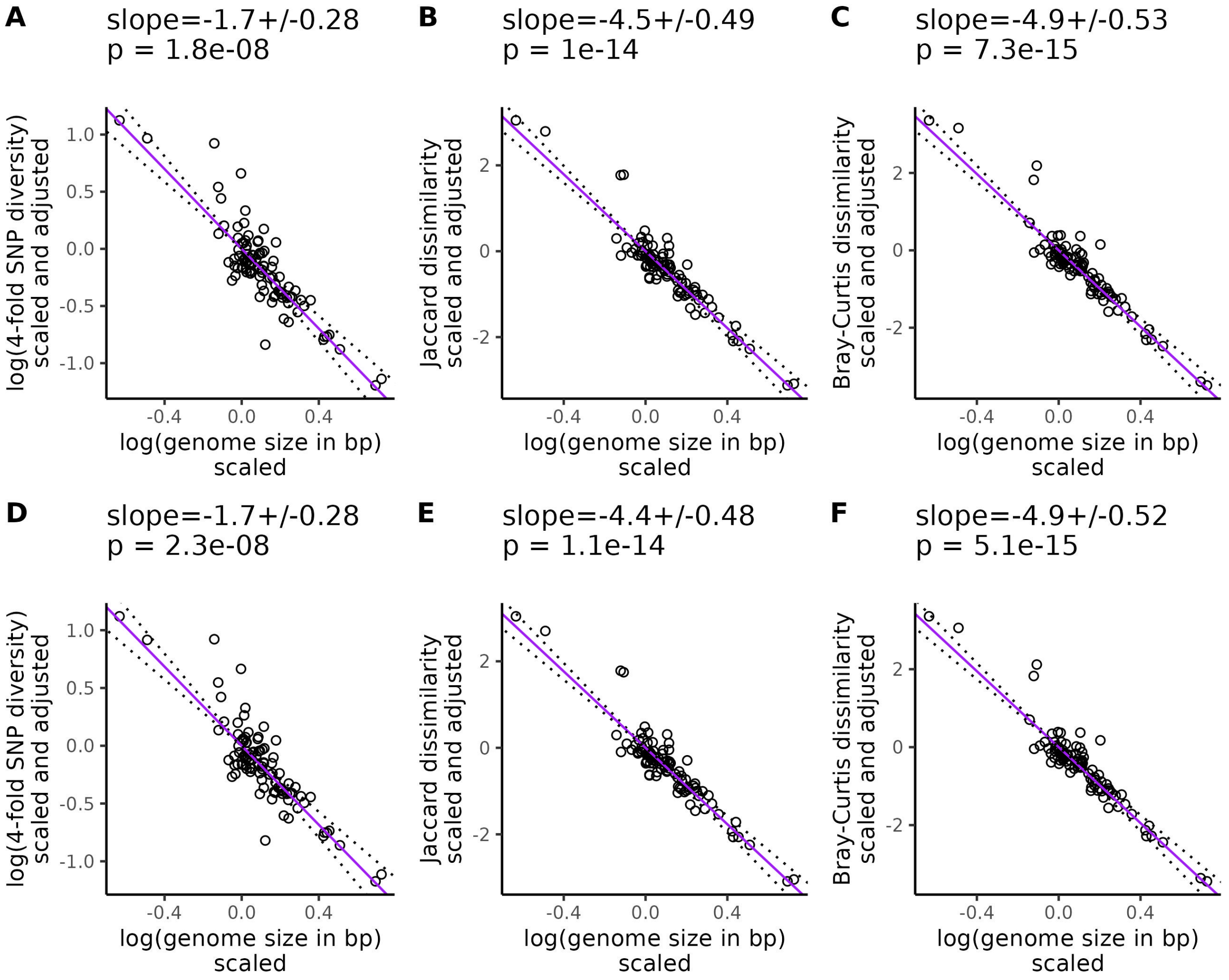

Figure S25: **Partial phylogenetic regression between diversity and genome size, controlling for GBIF range size.** Each point is a species and only species with > 0.5x mean coverage and > 1000 variant sites were included in the regression. The regressions are organized according to whether invaded ranges were excluded (A-C) or included (D-F) in the range size estimates. Lines give the relationship between the pairs of plotted variables after controlling for mating system, life cycle habit, cultivation status, range size, and evolutionary history. The statistics across the top of each plot give the value of the slope of the lines (± the standard error) and the p-value testing whether the slope differs from zero. Before fitting the line, each response variable was scaled to a standard normal distribution (mean = 0, variance = 1), then multiplied by the inverse of the Cholesky decomposition of the phylogenetic variance-covariance matrix to correct for phylogenetic relationships. Dotted lines show the partial regression slope ± one standard error. All logarithms are base 10.

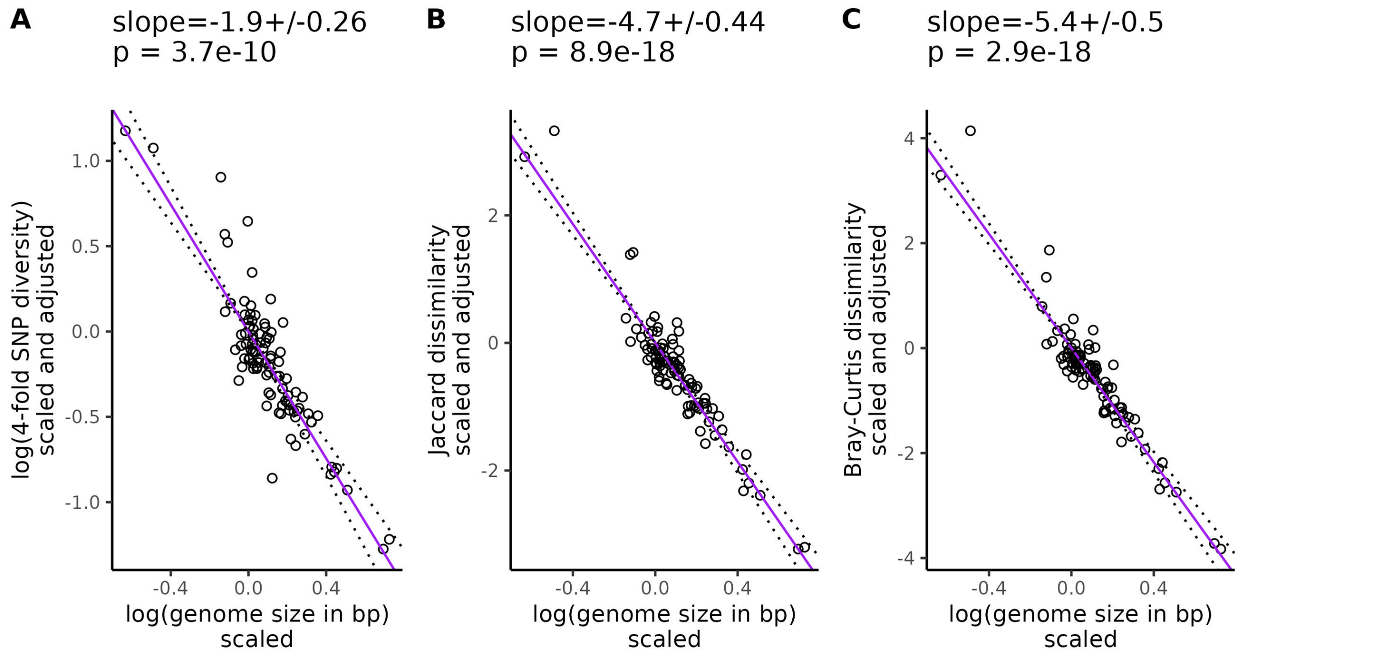

Figure S26: **Partial phylogenetic regression between diversity and genome size, controlling for height.** Each point is a species and only species with > 0.5x mean coverage and > 1000 variant sites were included in the regression. The regressions are organized according to whether invaded ranges were excluded (A-C) or included (D-F) in the range size-squared height ratio. Lines give the relationship between the pairs of plotted variables after controlling for mating system, life cycle habit, cultivation status, height, and evolutionary history. The statistics across the top of each plot give the value of the slope of the lines ($\pm$ the standard error) and the p-value testing whether the slope differs from zero. Before fitting the line, each response variable was scaled to a standard normal distribution (mean = 0, variance = 1), then multiplied by the inverse of the Cholesky decomposition of the phylogenetic variance-covariance matrix to correct for phylogenetic relationships. Dotted lines show the partial regression slope $\pm$ one standard error. All logarithms are base 10.

6 Supplemental Table captions

Table S1 contains the metadata for all of the datasets used in this study, including sources for genome assemblies, genome annotations, population-level sequencing datasets, and GBIF observations. Table S2 contains all of the covariate and response variable values used for fitting our phylogenetic least squares models. Table S3 contains the estimated coefficients of all of our phylogenetic least squares models and their related statistics, including p-values and standard errors. Table S4 contains the model-level statistics for each phylogenetic least squares model, including R^2 values and F-test results.
